# Physical Inactivity and Non-Communicable Disease Burden in Low-, Middle-, and High-Income Countries

**DOI:** 10.1136/bjsports-2020-103640

**Published:** 2021-03-29

**Authors:** Peter T. Katzmarzyk, Christine Friedenreich, Eric Shiroma, I-Min Lee

**Affiliations:** 1 Pennington Biomedical Research Center, 6400 Perkins Road, Baton Rouge, LA, 70810; 2 Cumming School of Medicine and Faculty of Kinesiology, University of Calgary, and Department of Cancer Epidemiology and Prevention Research, Cancer Care Alberta, Alberta Health Services, Room 514, Holy Cross Centre, 2210 2nd St SW, Calgary, AB, T2S 3C3; 3 Laboratory of Epidemiology and Population Science, National Institute on Aging, 7201 Wisconsin Ave, Gateway Bldg, Suite 2N300, Bethesda, MD 20814; 4 Department of Epidemiology, Harvard T.H. Chan School of Public Health, and Division of Preventive Medicine, Brigham and Women’s Hospital, 900 Commonwealth Avenue East, Boston, MA, 02215

**Keywords:** physical activity, exercise, disease burden, population attributable risk, chronic disease

## Abstract

**Objectives:**

Physical inactivity is a risk factor for premature mortality and several non-communicable diseases. The purpose of this study was to estimate the global burden associated with physical inactivity, and to examine differences by country income and region.

**Methods:**

Population-level, prevalence-based population attributable risks (PAR) were calculated for 168 countries to estimate how much disease could be averted if physical inactivity were eliminated. We calculated PARs (% cases attributable to inactivity) for all-cause mortality, cardiovascular disease mortality, and non-communicable diseases including coronary heart disease, stroke, hypertension, type 2 diabetes, dementia, depression, and cancers of the bladder, breast, colon, endometrium, esophagus, stomach, and kidney.

**Results:**

Globally, 7.2% and 7.6% of all-cause and cardiovascular disease deaths, respectively, are attributable to physical inactivity. The proportions of non-communicable diseases attributable to physical inactivity range from 1.6% for hypertension to 8.1% for dementia. There was an increasing gradient across income groups; PARs were more than double in high-income compared to low-income countries. However, 69% of total deaths and 74% of cardiovascular disease deaths associated with physical inactivity are occurring in middle-income countries, given their population size. Regional differences were also observed, with the PARs occurring in Latin America/Caribbean and high-income Western and Asia-Pacific countries, and the lowest burden occurring in Oceania and East/Southeast Asia.

**Conclusion:**

The global burden associated with physical inactivity is substantial. The relative burden is greatest in high-income countries; however, the greatest number of people (absolute burden) affected by physical inactivity are living in middle-income countries given the size of their populations.

## Introduction

Physical inactivity is an established risk factor for premature mortality and several non-communicable diseases.^1^ It has been estimated that in 2008, physical inactivity caused 6-10% of the cases of premature mortality, coronary heart disease, type 2 diabetes, breast cancer and colon cancer globally.^2^ Further, the physical inactivity-related health care costs associated with these non-communicable diseases was estimated to be $53.8 billion worldwide in 2013, of which $31 billion was paid by the public sector.^3^ Despite the widespread acknowledgement of physical activity as a health enhancing behaviour, globally 27.5% of adults fail to meet the current public health guidelines for physical activity.^4^ Physical activity has the potential to contribute to achieving many of the United Nations’ Sustainable Development Goals (SDGs) for 2030.^5^ For example, increases in physical activity can contribute directly to SDG 3 (good health and wellbeing) by reducing premature mortality from non-communicable diseases.

As described above, the global impact of physical inactivity on all-cause mortality and a select number of non-communicable diseases (coronary heart disease, type 2 diabetes, breast cancer and colon cancer) has been described.^2^ However, in the last decade a large volume of research has clearly shown that physical inactivity impacts additional non-communicable diseases beyond these.^1^

There is great global variability in the prevalence and trends in physical inactivity. For example, the prevalence of physical inactivity in 2016 was more than double in high-income countries (36.8%) compared to low-income countries (16.2%), and the prevalence of physical inactivity increased in high-income countries between 2001 and 2016.^4^ Large-scale shifts in noncommunicable disease burden are also occurring, such that 80% of global noncommunicable disease deaths are now occurring in low-and middle-income countries.^6^ Therefore, the purpose of this study was to estimate the current global non-communicable disease burden associated with physical inactivity, and to examine differences by geographic region and level of country income.

## Methods

We first identified the major health outcomes associated with physical inactivity. To do this analysis, we relied on the evidence presented by the U.S. 2018 Physical Activity Guidelines Advisory Committee.^1^ Based on exhaustive literature searches, the Committee reported that there was strong evidence that physical inactivity was associated with increased risk of all-cause mortality, cardiovascular disease mortality, and incidence of coronary heart disease, stroke, hypertension, type 2 diabetes, several cancers (bladder, breast, colon, endometrial, esophageal, gastric, renal), dementia, and depression. The Committee considered the evidence for each particular outcome to be “strong” based on an assessment of the magnitude and precision of the effect, the quantity and consistency of the results, risk of bias or study limitations, the generalizability of the results to the U.S. population, and the applicability of the study populations, exposures and outcomes to address the question.^1^ To be conservative, we did not include outcomes that the Committee deemed to have moderate, weak or insufficient evidence for associations with physical inactivity.

We used a population-level approach to estimate the global health burden associated with physical inactivity. As such, we computed a “semi-adjusted Population Attributable Risk (PAR_semi_)” for each outcome that uses the formula for calculating crude PAR but employs a multivariable adjusted RR, which has been shown to have low relative bias.^7^ That is, PAR_semi_ was calculated as [P(RR–1)]/[1 + P(RR–1)], where P is the prevalence of physical inactivity in the source population and RR is the multivariable-adjusted relative risk for the outcome in a physically inactive person.^7^ The PAR_semi_ provides a theoretical estimate of the proportion of an outcome that is attributable to a given exposure, in this case, physical inactivity.

We obtained the prevalence of insufficient physical activity from a recent publication of data collected in 2016 for adults from 168 countries.^4^ Insufficient physical activity was defined as not meeting current guidelines (doing at least 150 min of moderate-intensity, or 75 min of vigorous-intensity physical activity per week, or any equivalent combination of the two). The data were collected using primarily the Global Physical Activity Questionnaire (GPAQ) or the International Physical Activity Questionnaire (IPAQ), and included physical activity at work, at home, for transport, and during leisure time.^4^ Given that the IPAQ is known to over-report physical activity, the prevalence estimates were adjusted for this over-estimation using regression procedures.^4^ For outcomes restricted to women (breast cancer and endometrial cancer), we used the prevalence of insufficient physical activity for women only.

We obtained summary RR estimates for the effects of physical inactivity on each outcome from our original publication on physical inactivity and noncommunicable disease burden,^2^ meta-analyses identified by the U.S. 2018 Physical Activity Guidelines Advisory Committee^1^ and the updated searches conducted by the World Health Organization Physical Activity Guidelines Development Group (searches conducted up to September 2019).^8^ To ensure that we included the most up-to-date evidence, we conducted updated searches of Pubmed (January 1, 2018 through March 25, 2020) for meta-analyses and pooled analyses. The search terms included keywords related to physical activity (physical activity, exercise, motor activity, and walking) and the health outcomes of interest. The search resulted in 1929 potential papers, of which seven were deemed relevant to this study and were included in the list of meta-analyses and pooled analyses for potential inclusion in the final list.

From the compiled set of meta-analyses and pooled analyses, we chose the largest and most recently published meta-analysis or pooled analysis for each outcome that provided a RR for the contrast between low activity (or none) and a level of activity that approximated meeting the physical activity guidelines (7.5 to 14.9 MET-h/week), or moderate levels of activity (see Table 1). All meta-analyses and pooled analyses estimated RRs adjusted for potential confounders (meta-analyses generally used maximally adjusted RRs from the individual studies).

We computed the PAR_semi_ for each outcome, by country as well as by World Bank Income Classification (2016; low, middle, and high)^9^ and geographic region. We used Monte Carlo simulation techniques (10 000 simulations) to estimate 95% CIs. We assumed normal distributions for physical inactivity prevalence and the log of the RRs. Given that single RR estimates were used to compute all PARs for a given outcome, differences in PARs across countries or regions reflect the differences in physical inactivity prevalence. To estimate the number of global deaths attributable to physical inactivity, we applied the PARs for all-cause mortality and CVD mortality to the number of deaths (ages ≥15 y) from low-, middle-and high-income countries in 2016.^10^

### Patient and Public Involvement

No patients were involved in the planning, design, or research idea for this systematic review. Nor were they involved in the analyses or data collection for the work. The results from the present study will be disseminated through institutional websites and press releases.

## Results

The largest and most recent meta-analyses and pooled analyses that examined the associations between physical inactivity and the 15 health outcomes identified as having strong evidence of association are shown in Supplementary Table 1.^1^ One meta-analysis was from our original study of physical activity and global noncommunicable disease burden,^2^ while two meta-analyses (covering 4 outcomes) were identified in the 2018 U.S. Physical Activity Guidelines Advisory Committee.^11,12^ One additional meta-analysis was identified through the searches of the World Health Organization Physical Activity Guidelines Development Group,^13^ while two additional meta-analyses and one pooled analysis (all cancer outcomes) were identified by our updated search through March 2020.^14–16^

Table 1 presents the summary relative risk estimates from the selected meta-analyses along with the global PARs. A total of 7.2% and 7.6% of all-cause and cardiovascular disease deaths, respectively, are attributable to physical inactivity. Further, the proportions of non-communicable diseases attributable to physical inactivity range from 1.6% for hypertension to 8.1% for dementia.

**Figure 1.**
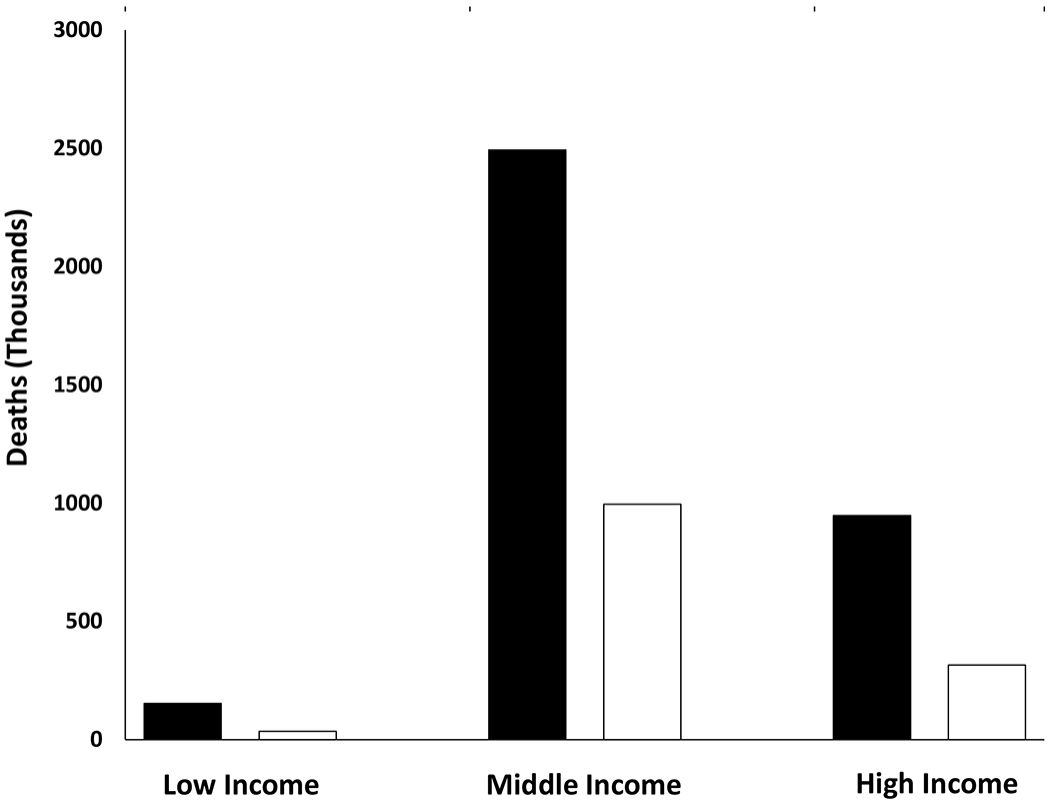
Number of total (black bars) and cardiovascular disease (white bars) deaths in low-, middle- and high-income countries attributable to physical inactivity, 2016.

Table 2 presents the PARs for each outcome by World Bank Income group. There is a clear gradient of increasing PARs across low-to middle-to high-income countries for each outcome. The PARs for all outcomes in high-income countries are more than double those of low-income countries. Figure 1 presents the total number of all-cause and cardiovascular disease deaths in low-, middle-and high-income countries. Despite higher PARs in high-income countries, 69% of total deaths and 74% of cardiovascular disease deaths associated with physical inactivity are occurring in middle-income countries because of the greater population numbers living in these countries.

Table 3 presents the PARs by geographic region. In general, the highest non-communicable disease burden associated with physical inactivity is in Latin American and Caribbean countries, and high income Western and Asia Pacific countries, followed by countries in Central Asia, the Middle East and North Africa. Countries in Sub-Saharan Africa, Oceania, and East and Southeast Asia have the lowest non-communicable disease burden associated with physical inactivity. Country-specific PARs for each of the outcomes are presented in Supplementary Tables 1–4.

## Discussion

### Statement of principal findings

The results here clearly demonstrate that physical inactivity is responsible for a significant global health burden: more than 7% of all-cause and cardiovascular disease deaths and up to 8% of non-communicable diseases are attributable to physical inactivity. These data provide a robust update of an earlier analysis that included only five outcomes linked to physical inactivity at the time,^2^ and include 15 outcomes that have strong evidence for associations with physical inactivity. Estimates of PARs for risk factors vary widely based on methodologic differences across studies. However, to contextualize our results, the 7% global PAR for all-cause mortality associated with physical inactivity compares with an estimated global PAR of 8.7% for global tobacco use,^17^ 1.2% for global sugar-sweetened beverage consumption,^18^ 10% in women and 11% in men for obesity in Europe,^19^ and between 3% to 15% for obesity in the United States.^20^

### Strengths and weaknesses

This study has several strengths including the use of summary relative risk estimates from the largest and most-recent meta-analyses and pooled analyses available. Since we only included outcomes that had a “strong” level of evidence of association based on the U.S. 2018 Physical Activity Guidelines Advisory Committee,^1^ and the updated review done by the World Health Organization Physical Activity Guideline Development Group, these estimates are likely conservative. These two review committees identified many other health outcomes that also are associated with physical activity but currently only have moderate or lower levels of evidence for an association with physical activity. Hence, the tiue magnitude of the burden of non-communicable disease that could be attributed to physical inactivity is likely considerably greater than what we have estimated here. Finally, we were also able to provide estimates for 168 low-, middle- and high-income countries that had prevalence data available, which represents 96% of the world’s population.^4^

The study weaknesses include the fact that the population-based approach that we used to estimate the disease burden results are theoretical. We used the PAR_semi_, which has been shown to have low relative bias;^7^ however, it is unknown how much bias was introduced by using this method in the current analysis. When relying on PARs to estimate burden, competing risks are not accounted for, which may bias the PAR estimates.^21^ This lack of adjustment for competing risks may have had an impact on our PAR estimates given that physical inactivity is more strongly associated with mortality than with the non-communicable diseases of interest. The same RR estimates were applied to all countries rather than using country-specific estimates; however, most of the evidence of associations come from studies conducted in high-income countries. While this approach is a limitation, it also standardizes comparisons across countries, and serves as a starting point for countries and regions to build context-specific evidence on associations between physical inactivity and health outcomes. Furthermore, we attempted to select RR estimates comparing physical inactivity versus moderate levels of activity (or meeting guidelines; ~7.5 to 14.9 MET-h/week). While the reference group was quite consistent, some studies reported “low” levels of activity and others reported “none” as the comparator, which may introduce some error into our PAR_semi_ estimates. Unfortunately, we cannot overcome this limitation based on the data available to us. In a similar manner, we were unable to estimate PARs using a dose-response approach (i.e., modelling the risk at multiple levels of physical inactivity, given that the global prevalence data are presented using a dichotomous variable).^4^ Furthermore, we were unable to account for different exposure-outcome associations at different ages; given different age distributions across countries, these differences could have an impact on the differential burden associated with physical inactivity in different countries. We relied on prevalence data from a published study that primarily used data from the GPAQ and IPAQ; although the analysis adjusted for over-reporting in the IPAQ, it is unknown how much of the variability in prevalence observed by income and region is explained by unmeasured factors.

### Meaning of the study

There is an increasing gradient in the burden of physical inactivity across low- to middle- to high-income countries for each outcome which reflects the underlying prevalence of physical inactivity. While country income classification and human development index are different constmcts related to development, our results are congruent with those from an earlier study which showed a weak positive association (rho = 0.27) between the prevalence of physical inactivity and human development index across 76 countries.^22^ Although not directly comparable to our results, Strain and colleagues recently reported that the existing physical activity prevalence was averting more deaths in low-income countries than in high income countries, which is consistent with our overall interpretation.^23^ On the other hand, the greatest absolute number of deaths attributable to physical inactivity occurred in middle-income countries (Figure 1). This higher number reflects the larger population numbers living in middle-income countries and hence a greater number of total deaths. For example, in 2016, 3.6 M, 36.7 M, and 10.2 M deaths occurred among the population that was ≥15 years old in low-, middle- and high-income countries, respectively.^10^ Thus, the evidence indicates that the greatest relative burden associated with physical inactivity occurs in high-income countries; however, the greatest absolute burden is occurring in middle-income countries.

We relied on the largest, most recent meta-analyses and pooled analyses available to estimate the appropriate summary RRs associated with physical inactivity. All meta-analyses were published within the last 9 years; with the oldest being for all-cause mortality. The summary RR estimates from these studies have been confirmed by other published analyses. For example, the RRs for colon cancer and breast cancer from the pooled analysis used in the present study are 1.11 (1.03 to 1.19) and 1.09 (1.03 to 1.15) respectively, which are similar to those reported in a meta-analysis of cohort studies (1.11 [1.05 to 1.18] and 1.03 [1.00 to 1.07). Further, the summary RR for all-cause mortality used in the present study is 1.28 (1.21 to 1.36);^2^ which is somewhat lower than a recent pooled analysis of six prospective cohort studies (1.45; 1.43 to 1.49),^24^ making our estimates conservative.

### Unanswered questions and future research

Future large-scale studies that track actual changes in physical activity over time using device-based measurement methods and resulting changes in non-communicable disease incidence and prevalence will provide more definitive results. Furthermore, future studies should incorporate surveillance and outcome data across several levels of physical activity to improve the quantification of the global burden across the physical activity spectrum. In a similar manner, data on physical activity across sex and age groups should be incorporated to improve the estimates of disease burden across countries that have different sex and age distributions.

## Conclusion

The global health burden associated with physical inactivity is substantial. These data are crucial to inform governments and policy makers, particularly in populous middle-income countries. While the relative non-communicable disease burden is greatest in high-income countries, middle-income countries have the greatest number of people affected by physical inactivity. In 2018, the World Health Assembly (WHA) approved the *“Global Action Plan on Physical Activity 2018–2030”* and adopted a target to reduce global levels of physical inactivity by 15% by 2030.^25^ Our results provide evidence that the public health burden associated with physical inactivity is truly a global issue that will require international collaboration to mobilize change and achieve these public health goals.

## Supplementary Material

Supp1

## Figures and Tables

**Table 1.** Source Studies, Summary Relative Risks for Physical Inactivity, and Associated Global Population Attributable Risks

Outcome/Study	Study Design	Comparisons for Summary Relative Risk	Summary Relative Risk (95% CI)	PAR_semi_(95% CI)
All-Cause Mortality Lee et al. 2012^2^	Meta-analysis of 32 prospective cohort studies	Low versus moderate leisure-time physical activity	1.28 (1.21 to 1.36)	7.2 (5.4 to 9.0)
Cardiovascular Disease Mortality Cheng et al. 2018^14^	Meta-analysis of 40 prospective cohort studies	Low versus moderate recreational physical activity	1.30(1.23 to 1.35)	7.6 (6.1 to 9.3)
Dementia Guure et al. 2017^15^	Meta-analysis of 25 prospective cohort studies	Lowest versus moderate levels of physical activity	1.32 (1.06 to 1.64)	8.1 (2.6 to 14.9)
Depression Schuch et al. 2018^13^	Meta-analysis of 4 prospective cohort studies	Lowest versus 150 minutes of moderate-to-vigorous physical activity per week	1.28 (1.01 to 1.62)	7.2 (1.3 to 14.5)
Coronary Heart Disease Kyu et al. 2016^11^	Meta-analysis of 43 prospective cohort studies	<600 MET-min/week versus 600–3999 MET-min/week of total physical activity across all domains	1.19 (1.13 to 1.26)	5.0 (3.5 to 6.5)
Stroke Kyu et al. 2016^11^	Meta-analysis of 26 prospective cohort studies	<600 MET-min/week versus 600–3999 MET-min/week of total physical activity across all domains	1.19 (1.09 to 1.28)	5.0 (2.9 to 7.3)
Type 2 Diabetes Kyu et al. 2016^11^	Meta-analysis of 55 prospective cohort studies	<600 MET-min/week versus 600–3999 MET-min/week of total physical activity across all domains	1.17 (1.11 to 1.23)	4.5 (3.1 to 6.0)
Hypertension Liu et al. 2017^12^	Meta-analysis of 24 prospective cohort studies	None versus 10 MET-h/week of leisure-time physical activity	1.06(1.03 to 1.09)	1.6 (1.0 to 2.4)
Bladder Cancer Matthews et al. 2020^16^	Pooled analysis of data from 9 prospective cohorts	None versus 7.5 – 14.9 MET-h/week of leisure-time physical activity.	1.08(0.93 to 1.25)	2.2 (−0.3 to 16.2)
Breast Cancer* Matthews et al. 2020^16^	Pooled analysis of data from 9 prospective cohorts	None versus 7.5 – 14.9 MET-h/week of leisure-time physical activity.	1.09(1.03 to 1.15)	2.8 (1.2 to 4.4)
Colon Cancer Matthews et al. 2020^16^	Pooled analysis of data from 9 prospective cohorts	None versus 7.5 – 14.9 MET-h/week of leisure-time physical activity.	1.11 (1.03 to 1.19)	2.9 (1.2 to 4.9)
Endometrial Cancer* Matthews et al. 2020^16^	Pooled analysis of data from 9 prospective cohorts	None versus 7.5 – 14.9 MET-h/week of leisure-time physical activity.	1.09 (0.96 to 1.22)	2.8 (−0.4 to 6.4)
Esophaeeal Cancer Matthews et al. 2020^16^	Pooled analysis of data from 9 prospective cohorts	None versus 7.5 – 14.9 MET-h/week of leisure-time physical activity.	1.28 (0.85 to 1.96)	7.2 (−2.3 to 20.9)
Gastric Cancer Matthews et al. 2020^16^	Pooled analysis of data from 9 prospective cohorts	None versus 7.5 – 14.9 MET-h/week of leisure-time physical activity.	1.27 (0.93 to 1.69)	6.9 (−0.3 to 16.2)
Renal Cancer Matthews et al. 2020^16^	Pooled analysis of data from 9 prospective cohorts	None versus 7.5 – 14.9 MET-h/week of leisure-time physical activity.	1.28 (1.06 to 1.54)	7.2 (2.4 to 12.9)

The global prevalence of insufficient physical activity of 27.5% (95% CI: 25.0 to 32.2) was applied to compute the PAR_semi_.

* The global prevalence of insufficient physical activity among women of 31.7% (95% CI: 28.6 to 39.0) was applied to compute the PAR_semi_ for breast cancer and endometrial cancer.

**Table 2.** Prevalence and Population Attributable Risks Associated with Physical Inactivity in Low-, Middle-, and High-Income Countries

	Low- Income	Middle- Income	High- Income
Prevalence (95% CI)^*,ǂ^	16.2 (14.2 to 17.9)	26.0 (22.6 to 31.8)	36.8 (35.0 to 38.0)
PAR_semi_ (95% CI)			
All-Cause Mortality	4.3 (3.3 to 5.5)	6.8 (5.0 to 8.7)	9.3 (7.2 to 11.6)
CVD Mortality	4.6 (3.8 to 5.6)	7.2 (5.7 to 9.0)	9.9 (8.2 to 11.7)
Coronary Heart Disease	3.0 (2.1 to 3.9)	4.7 (3.3 to 6.3)	6.5 (4.7 to 8.5)
Stroke	3.0 (1.8 to 4.4)	4.7 (2.7 to 7.0)	6.5 (3.8 to 9.4)
Hypertension	1.0 (0.6 to 1.4)	1.5 (0.9 to 2.2)	2.2 (1.3 to 3.1)
Type 2 Diabetes	2.7 (1.9 to 3.5)	4.2 (2.9 to 5.7)	5.9 (4.2 to 7.7)
Bladder Cancer	1.3 (−0.8 to 3.6)	2.0 (−1.2 to 5.7)	2.9 (−1.5 to 7.9)
Breast Cancer^*^	1.7 (0.8 to 2.6)	2.6 (1.2 to 4.2)	3.6 (1.6 to 5.7)
Colon Cancer	1.8 (0.7 to 2.9)	2.8 (1.1 to 4.6)	3.9 (1.6 to 6.4)
Endometrial Cancer^*^	1.7 (−0.2 to 3.8)	2.6 (−0.4 to 6.1)	3.6 (−0.6 to 8.3)
Esophageal Cancer	4.3 (−1.5 to 12.8)	6.8 (−2.4 to 20.1)	9.3 (−3.2 to 27.5)
Gastric Cancer	4.2 (−0.2 to 9.9)	6.6 (−0.2 to 15.3)	9.0 (−0.4 to 21.1)
Renal Cancer	4.3 (1.4 to 7.8)	6.8 (2.3 to 12.4)	9.3 (3.2 to 16.6)
Dementia	4.9 (1.5 to 9.2)	7.7 (2.4 to 14.3)	10.5 (3.3 to 19.5)
Depression	4.3 0.8 to 8.8)	6.8 (1.2 to 13.6)	9.3 11.6 to 18.7)

* The prevalence of insufficient physical activity among women (low-income: 18.8 %; 95% CI: 15.9 to 21.4; middle-income: 30.1%; 95% CI 26.0 to 39.5; high-income: 41.6%; 95% CI: 39.1 to 43.9) was applied to compute the PAR_semi_ for breast cancer and endometrial cancer.

ǂ Prevalence estimates were obtained from Guthold et al.^4^

**Table 3.** Population Attributable Risks Associated with Physical Inactivity in Different Geographical Regions

Outcome	Central Asia, Middle East, & North Africa	Central & Eastern Europe	East & Southeast Asia	High-Income Asia Pacific	High-Income Western Countries	Latin America & Caribbean	Oceania	South	Sub-Saharan Africa
Prevalence (95% CI)^*,ǂ^	32.8 (31.0 to 32.2)	23.4 (20.9 to 28.0)	17.3 (15.8 to 22.1)	35.7 (34.4 to 37.0)	36.8 (34.6 to 38.4)	39.1 (37.8 to 40.6)	16.3 (14.3 to 20.7)	33.0 (23.0 to 51.7)	21.4(19.1 to 23.3)
PAR_semi_ (95% CI)									
All-Cause Mortality	8.4 (6.5 to 10.4)	6.2 (4.6 to 7.8)	4.6 (3.4 to 6.0)	9.1 (7.0 to 11.2)	9.3 (7.2 to 11.5)	9.9 (7.7 to 12.2)	4.4 (3.2 to 5.6)	8.5 (5.0 to 12.3)	5.7 (4.3 to 7.1)
CVD Mortality	9.0 (7.4 to 10.6)	6.6 (5.2 to 8.0)	4.9 (3.9 to 6.1)	9.7 (8.1 to 11.4)	9.9 (8.3 to 11.7)	10.5 (8.7 to 12.4)	4.7 (3.6 to 5.8)	9.0 (5.5 to 12.8)	6.0 (3.9 to 7.2)
Coronary Heart Disease	5.9 (4.2 to 7.6)	4.3 (3.0 to 5.7)	3.2 (2.2 to 4.3)	6.4 (4.6 to 8.2)	6.5 (4.7 to 8.4)	6.9 (5.0 to 8.9)	3.0 (2.1 to 4.1)	5.9 (3.3 to 9.0)	3.9 (2.8 to 5.2)
Stroke	5.9 (3.5 to 8.5)	4.3 (2.5 to 6.2)	3.2 (1.8 to 4.7)	6.4 (3.8 to 9.2)	6.5 (3.9 to 9.4)	6.9 (4.1 to 10.0)	3.0 (1.7 to 4.4)	5.9 (2.9 to 9.6)	3.9 (2.2 to 5.7)
Hypertension	1.9 (1.1 to 2.8)	1.4 (0.8 to 2.0)	1.0 (0.6 to 1.5)	2.1 (1.2 to 3.0)	2.2 (1.3 to 3.1)	2.3 (1.3 to 3.3)	1.0 (0.6 to 1.4)	1.9 (1.0 to 3.2)	1.3 (0.7 to 1.8)
Type 2 Diabetes	5.3 (3.8 to 6.9)	3.8 (2.7 to 5.1)	2.9 (2.0 to 3.9)	5.7 (4.0 to 7.5)	5.9 (4.2 to 7.7)	6.2 (4.5 to 8.1)	2.7 (1.8 to 3.7)	5.3 (3.0 to 8.0)	3.5 (2.5 to 4.6)
Bladder Cancer	2.6 (−1.4 to 7.1)	1.8 (−1.0 to 5.2)	1.4 (−0.8 to 3.8)	2.8 (−1.5 to 7.7)	2.9 (−1.7 to 7.9)	3.0 (−1.7 to 8.5)	1.3 (−0.7 to 3.6)	2.6 (−1.5 to 7.5)	1.7 (−0.9 to 4.7)
Breast Cancer^*^	3.5 (1.6 to 5.4)	2.2 (0.9 to 3.5)	1.5 (0.6 to 2.5)	3.3 (1.5 to 5.3)	3.7 (1.6 to 5.8)	3.8 (1.8 to 6.0)	1.8 (0.8 to 2.9)	3.7 (1.4 to 6.7)	2.2 (1.0 to 3.5)
Colon Cancer	3.5 (1.4 to 5.7)	2.5 (1.0 to 4.2)	1.9 (0.7 to 3.2)	3.8 (1.6 to 6.1)	3.9 (1.6 to 6.4)	4.1 (1.7 to 6.7)	1.8 (0.7 to 2.9)	3.5 (1.3 to 6.3)	2.3 (0.9 to 3.7)
Endometrial Cancer^*^	3.5 (−0.6 to 8.0)	2.2 (−0.3 to 5.1)	1.5 (−0.3 to 3.5)	3.3 (−0.5 to 7.6)	3.7 (−0.6 to 8.3)	3.8 (−0.6 to 8.7)	1.8 (−0.3 to 4.2)	3.7 (−0.6 to 9.3)	2.2 (−0.4 to 5.0)
Esophageal Cancer	8.4 (−3.0 to 24.4)	6.2 (−2.1 to 17.9)	4.6 (−1.7 to 13.7)	9.1 (−3.2 to 26.6)	9.3 (−3.3 to 27.7)	9.9 (−3.5 to 29.2)	4.4 (−1.6 to 12.9)	8.5 (−2.9 to 25.9)	5.7 (−2.0 to 16.6)
Gastric Cancer	8.1 (−0.3 to 19.0)	5.9 (−0.2 to 14.2)	4.5 (−0.2 to 10.6)	8.8 (−0.6 to 20.5)	9.0 (−0.4 to 21.6)	9.6 (−0.5 to 22.3)	4.2 (−0.2 to 10.1)	8.2 (−0.3 to 20.3)	5.5 (−0.2 to 12.8)
Renal Cancer	8.4 (2.8 to 14.9)	6.2 (2.1 to 11.1)	4.6 (1.6 to 8.3)	9.1 (3.0 to 16.3)	9.3 (3.1 to 16.8)	9.9 (3.4 to 17.4)	4.4 (1.4 to 7.9)	8.5 (2.5 to 16.2)	5.7 (1.8 to 10.2)
Dementia	9.5 (2.8 to 17.5)	7.0 (2.2 to 12.9)	5.3 (1.6 to 9.7)	10.3 (3.2 to 18.8)	10.5 (3.3 to 19.1)	11.1 (3.6 to 20.4)	5.0 (1.5 to 9.3)	9.6 (2.6 to 19.1)	6.4 (2.0 to 11.8)
Depression	8.4 (1.5 to 16.9)	6.2 (1.0 to 12.4)	4.6 (0.8 to 9.4)	9.1 (1.6 to 18.4)	9.3 (1.5 to 18.7)	9.9 (1.7 to 20.0)	4.4 (0.8 to 9.0)	8.5 (1.3 to 18.1)	5.7 (1.0 to 11.4)

* The prevalence of insufficient physical activity among women (Central Asia, Middle East & North Africa: 39.9 %; 95% CI: 37.9 to 42.7; Central & Eastern Europe: 24.7%; 95% CI: 21.7 to 33.9; East & Southeast Asia: 16.9%; 95% CI: 14.9 to 25.7; High-Income Asia Pacific: 38.3%; 95% CI: 37.4 to 42.6; High-Income Western: 42.3%; 95% CI: 39.1 to 45.4; Latin America & Caribbean: 43.7%; 95% CI: 42.9 to 46.5; Oceania: 203.%; 95% CI: 18.8 to 28.7; South Asia: 43.0%; 95% CI: 29.6 to 74.9; Sub-Saharan Africa: 24.8%; 95% CI: 21.8 to 27.2) was applied to compute the PAR_semi_ for breast cancer and endometrial cancer.

ǂ Prevalence estimates were obtained from Guthold et al.^4^

## What are the New Findings?

This study provides the most complete description of the global non-communicable disease burden associated with physical inactivity and presents results by level of country income and by geographic region. Physical inactivity is responsible for up to 8% of deaths and non-communicable diseases that are attributable to physical inactivity. There is an increasing gradient of relative burden with increasing country income; however, middle-income countries have the greatest number of people affected by physical inactivity because of their larger population size.

## How Might it Impact on Clinical Practice in the Near Future?

Our results indicate that the relative non-communicable disease burden is greatest in high-income countries, while middle-income countries have the greatest number of people affected by physical inactivity because of their greater population size. These results emphasize the significant public health impact of physical inactivity on non-communicable diseases, which indicates that the promotion of physical activity should be emphasized in clinical practice in addition to public health efforts.
