# Supplementary material for: Physical Inactivity and Non-Communicable Disease Burden in Low-, Middle-, and High-Income Countries
Source: Br J Sports Med. Author manuscript; Available in PMC 2022 Jul 1. (PMC8478970; doi:10.1136/bjsports-2020-103640)
Supplement: Supp1 [file NIHMS1697438-supplement-Supp1.pdf]

Supplementary Appendix

Physical Inactivity and Non-Communicable Disease Burden in Low-, Middle-, and High-Income Countries

Peter T. Katzmarzyk, PhD[1], Christine Friedenreich, PhD[2], Eric Shiroma, ScD[3] and I-Min Lee, ScD[4]

[1]Pennington Biomedical Research Center, 6400 Perkins Road, Baton Rouge, LA, 70810

[2]Cumming School of Medicine and Faculty of Kinesiology, University of Calgary, and Department of Cancer Epidemiology and Prevention Research, Cancer Care Alberta, Alberta Health Services, Room 514, Holy Cross Centre, 2210 2nd St SW, Calgary, AB, T2S 3C3

[3]Laboratory of Epidemiology and Population Science, National Institute on Aging, 7201 Wisconsin Ave, Gateway Bldg, Suite 2N300, Bethesda, MD 20814

[4]Department of Epidemiology, Harvard T.H. Chan School of Public Health, and Division of Preventive Medicine, Brigham and Women's Hospital, 900 Commonwealth Avenue East, Boston, MA, 02215

Address for Correspondence:
Peter T. Katzmarzyk, PhD, Pennington Biomedical Research Center, 6400 Perkins Road, Baton Rouge, LA. 70808-4124; Phone (225) 763-2536; Fax: (225) 763-2927;
Email: Peter.Katzmarzyk@pbrc.edu

Supplementary Table 1. Population Attributable Risks for Physical Inactivity and All-Cause Mortality, Cardiovascular Disease Mortality, Coronary Heart Disease, Stroke, and Hypertension by Country

Country	All-Cause Mortality	Cardiovascular Disease Mortality	Coronary Heart Disease	Stroke	Hypertension
Algeria	8.6 (6.1 to 11.4)	9.2 (6.8 to11.8	6.0 (4.0 to 8.3)	6.0 (3.4 to 9.0)	2.0 (1.1 to3.0)
American Samoa	13.0 (9.4 to 17.1)	13.8 (10.4 to 17.5)	9.2 (6.2 to 12.6)	9.2 (5.3 to 13.9)	3.1 (1.7 to 4.6)
Andorra	9.7 (7.1 to 12.6)	10.3 (8.0 to 13.0)	6.8 (4.7 to 9.2)	6.8 (3.9 to 10.1)	2.3 (1.3 to 3.3)
Argentina	10.4 (7.6 to 13.5)	11.1 (8.5 to 13.8)	7.3 (5.0 to 9.9)	7.3 (4.2 to 10.9)	2.4 (1.4 to 3.6)
Armenia	6.0 (4.0 to 8.1)	6.4 (4.5 to 8.5)	4.1 (2.6 to 5.8)	4.1 (2.2 to 6.3)	1.3 (0.7 to 2.1)
Australia	7.8 (5.7 to 10.4)	8.4 (6.3 to 10.6)	5.5 (3.7 to 7.5)	5.5 (3.1 to 8.1)	1.8 (1.0 to 2.7)
Austria	7.8 (5.7 to 10.1)	8.3 (6.4 to 10.5)	5.4 (3.7 to 7.3)	5.4 (3.1 to 8.0)	1.8 (1.0 to 2.6)
Bahamas	10.8 (8.0 to 14.0)	11.5 (8.9 to 14.4)	7.6 (5.2 to 10.3)	7.6 (4.4 to 11.3)	2.5 (1.4 to 3.7)
Bangladesh	7.2 (4.4 to10.4)	7.7 (4.9 to 10.8)	5.0 (2.9 to 7.5)	5.0 (2.6 to 8.1)	1.6 (0.8 to 2.6)
Barbados	10.7 (7.9 to 13.9)	11.4 (8.7 to 14.4)	7.5 (5.2 to 10.3)	7.5 (4.3 to 11.2)	2.5 (1.4 to 3.7)
Belarus	3.8 (2.5 to 5.3)	4.1 (2.7 to 5.5)	2.6 (1.7 to 3.8)	2.6 (1.4 to 4.0)	0.8 (0.5 to 1.3)
Belgium	9.1 (6.7 to 11.8)	9.7 (7.6 to 12.0)	6.4 (4.4 to 8.5)	6.4 (3.6 to 9.4)	2.1 (1.2 to 3.1)
Benin	4.3 (3.0 to 5.7)	4.6 (3.3 to 5.9)	2.9 (1.9 to 4.1)	2.9 (1.6 to 4.5)	0.9 (0.5 to 1.4)
Bermuda	6.8 (4.7 to 9.3)	7.3 (5.2 to 9.6)	4.7 (3.1 to 6.7)	4.7 (2.6 to 7.2)	1.6 (0.8 to 2.3)
Bhutan	6.1 (4.4 to 7.8)	6.5 (5.0 to 8.0)	4.2 (2.9 to 5.6)	4.2 (2.4 to 6.2)	1.4 (0.8 to 2.0)
Bosnia & Herzegovina	6.7 (4.6 to 9.1)	7.1 (5.1 to 9.4)	4.6 (3.0 to 6.5)	4.6 (2.5 to 7.2)	1.5 (0.8 to 2.3)
Botswana	5.7 (4.1 to 7.6)	6.1 (4.6 to 7.9)	4.0 (2.7 to 5.4)	4.0 (2.2 to 5.9)	1.3 (0.7 to 1.9)
Brazil	11.6 (8.7 to 14.9)	12.4 (9.7 to 15.2)	8.2 (5.8 to 11.0)	8.2 (4.8 to 12.1)	2.7 (1.6 to 4.1)
British Virgin Islands	7.1 (5.0 to 9.5)	7.6 (5.6 to 9.7)	5.0 (3.3 to 6.9)	5.0 (2.8 to 7.5)	1.6 (0.9 to 2.4)
Brunei Darussalam	7.1 (4.6 to 9.9)	7.6 (5.1 to 10.4)	4.9 (3.0 to 7.2)	4.9 (2.6 to 7.8)	1.6 (0.8 to 2.5)
Bulgaria	9.8 (7.1 to 12.7)	10.4 (7.9 to 13.1)	6.8 (4.7 to 9.3)	6.8 (3.9 to 10.2)	2.3 (1.2 to 3.4)
Burkina Faso	5.4 (3.8 to 7.2)	5.7 (4.2 to 7.4)	3.7 (2.5 to 5.2)	3.7 (2.1 to 5.7)	1.2 (0.7 to 1.8)
Cabo Verde	5.2 (3.7 to 7.0)	5.6 (4.1 to 7.2)	3.6 (2.4 to 5.0)	3.6 (2.0 to 5.4)	1.2 (0.7 to 1.8)
Cambodia	2.9 (1.8 to 4.1)	3.1 (1.9 to 4.3)	2.0 (1.1 to 2.9)	2.0 (1.0 to 3.2)	0.6 (0.3 to 1.0)
Cameroon	7.4 (5.4 to 9.6)	7.9 (6.0 to 10.0)	5.1 (3.5 to 7.0)	5.1 (2.9 to 7.7)	1.7 (1.0 to 2.5)
Canada	7.4 (5.3 to 9.9)	7.9 (5.9 to 10.2)	5.2 (3.4 to 7.1)	5.2 (3.0 to 7.8)	1.7 (0.9 to 2.5)
Cayman Islands	7.5 (5.3 to 10.1)	8.0 (5.9 to 10.4)	5.2 (3.5 to 7.3)	5.2 (2.9 to 8.0)	1.7 (1.0 to 2.6)
Central African Republic	3.9 (2.7 to 5.2)	4.1 (3.0 to 5.3)	2.7 (1.7 to 3.7)	2.7 (1.5 to 4.0)	0.9 (0.5 to 1.3)
Chad	6.1 (4.2 to 8.2)	6.5 (4.7 to 8.6)	4.2 (2.8 to 6.0)	4.2 (2.3 to 6.5)	1.4 (0.8 to 2.1)
Chile	6.9 (4.9 to 9.2)	7.4 (5.4 to 9.6)	4.8 (3.2 to 6.7)	4.8 (2.7 to 7.3)	1.6 (0.9 to 2.4)
China	3.8 (2.5 to 5.2)	4.1 (2.8 to 5.4)	2.6 (1.7 to 3.7)	2.6 (1.4 to 4.0)	0.8 (0.5 to 1.3)
Colombia	11.0 (8.1 to 14.2)	11.7 (9.0 to 14.5)	7.7 (5.3 to 10.4)	7.7 (4.4 to 11.5)	2.6 (1.5 to 3.8)
Comoros	3.9 (2.7 to 5.1)	4.1 (3.1 to 5.3)	2.7 (1.8 to 3.7)	2.7 (1.5 to 4.0)	0.9 (0.5 to 1.3)
Congo	7.3 (5.1 to 9.7)	7.8 (5.8 to 10.0)	5.1 (3.4 to 6.9)	5.1 (2.9 to 7.5)	1.7 (0.9 to 2.5)
Cook Islands	4.9 (3.3 to 6.7)	5.3 (3.7 to 7.0)	3.4 (2.2 to 4.8)	3.4 (1.8 to 5.3)	1.1 (0.6 to 1.7)
Costa Rica	11.4 (8.5 to 14.7)	12.2 (9.5 to 15.0)	8.1 (5.6 to 10.9)	8.1 (4.6 to 11.9)	2.7 (1.6 to 3.9)
Cote d'Ivoire	8.5 (6.2 to 10.9)	9.0 (7.0 to 11.3)	5.9 (4.1 to 8.0)	5.9 (3.4 to 8.8)	2.0 (1.1 to 2.9)
Croatia	8.0 (5.7 to 10.6)	8.5 (6.4 to 10.9)	5.6 (3.8 to 7.7)	5.6 (3.1 to 8.4)	1.8 (1.0 to 2.7)
Cuba	9.5 (6.8 to 12.3)	10.0 (7.6 to 12.6)	6.6 (4.4 to 9.0)	6.6 (3.7 to 9.7)	2.2 (1.2 to 3.2)

Cyprus	11.1 (8.2 to 14.2)	11.8 (9.2 to 14.5)	7.8 (5.4 to 10.4)	7.8 (4.5 to 11.4)	2.6 (1.5 to 3.8)
Czech Republic	8.0 (5.7 to 10.6)	8.5 (6.5 to 10.8)	5.6 (3.7 to 7.7)	5.6 (3.2 to 8.3)	1.8 (1.0 to 2.7)
Democratic Republic of the Congo	6.3 (4.5 to 8.2)	6.7 (5.0 to 8.5)	4.3 (2.9 to 5.9)	4.3 (2.5 to 6.5)	1.4 (0.8 to 2.1)
Denmark	7.4 (5.3 to 9.6)	7.9 (6.0 to 9.9)	5.1 (3.5 to 7.0)	5.1 (2.9 to 7.7)	1.7 (1.0 to 2.5)
Dominica	5.7 (4.0 to 7.6)	6.1 (4.4 to 7.9)	3.9 (2.6 to 5.5)	3.9 (2.2 to 6.0)	1.3 (0.7 to 2.0)
Dominican Republic	9.8 (7.2 to 12.9)	10.5 (8.0 to 13.2)	6.9 (4.7 to 9.4)	6.9 (3.9 to 10.2)	2.3 (1.3 to 3.4)
Ecuador	7.1 (4.9 to 9.5)	7.5 (5.5 to 9.8)	4.9 (3.2 to 6.9)	4.9 (2.7 to 7.4)	1.6 (0.9 to 2.4)
Egypt	8.0 (5.7 to 10.6)	8.5 (6.2 to 11.0)	5.6 (3.7 to 7.7)	5.6 (3.1 to 8.4)	1.8 (1.0 to 2.8)
Eritrea	5.9 (4.5 to 7.5)	6.3 (5.0 to 7.7)	4.1 (2.9 to 5.5)	4.1 (2.4 to 6.0)	1.3 (0.8 to 1.9)
Estonia	8.2 (6.0 to 10.8)	8.8 (6.6 to 11.1)	5.7 (3.9 to 7.9)	5.7 (3.2 to 8.6)	1.9 (1.1 to 2.8)
Ethiopia	4,0 (3.0 to 5.2)	4.3 (3.3 to 5.3)	2.8 (1.9 to 3.7)	2.8 (1.6 to 4.1)	0.9 (0.5 to 1.3)
Fiji	4.7 (3.1 to 6.4)	5.0 (3.4 to 6.6)	3.2 (2.1 to 4.6)	3.2 (1.7 to 5.0)	1.0 (0.6 to 1.6)
Finland	4.4 (3.2 to 5.9)	4.7 (3.5 to 6.1)	3.1 (2.1 to 4.2)	3.1 (1.7 to 4.6)	1.0 (0.6 to 1.5)
France	7.6 (5.5 to 9.9)	8.1 (6.2 to 10.2)	5.3 (3.6 to 7.2)	5.3 (3.0 to 7.9)	1.7 (1.0 to 2.6)
French Polynesia	4.8 (3.2 to 6.6)	5.1 (3.6 to 6.8)	3.3 (2.1 to 4.7)	3.3 (1.8 to 5.0)	1.1 (0.6 to 1.6)
Gabon	6.6 (4.5 to 9.0)	7.1 (5.0 to 9.3)	4.6 (3.0 to 6.5)	4.6 (2.5 to 7.0)	1.5 (0.8 to 2.3)
Gambia	5.6 (3.9 to 7.5)	6.0 (4.4 to 7.7)	3.9 (2.5 to 5.3)	3.9 (2.2 to 5.8)	1.3 (0.7 to 1.9)
Georgia	4.8 (3.2 to 6.6)	5.1 (3.5 to 6.8)	3.3 (2.1 to 4.7)	3.3 (1.8 to 5.1)	1.1 (0.6 to 1.6)
Germany	10.6 (8.0 to 13.5)	11.2 (8.9 to 13.9)	7.4 (5.1 to 9.9)	7.4 (4.3 to 11.0)	2.5 (1.4 to 3.6)
Ghana	5.8 (4.2 to 7.4)	6.1 (4.7 to 7.7)	4.0 (2.7 to 5.4)	4.0 (2.3 to 5.9)	1.3 (0.7 to 1.9)
Greece	9.6 (7.1 to 12.3)	10.2 (7.9 to 12.7)	6.7 (4.7 to 9.0)	6.7 (3.9 to 10.0)	2.2 (1.3 to 3.3)
Grenada	7.4 (5.1 to 9.9)	7.9 (5.8 to 10.3)	5.2 (3.4 to 7.2)	5.2 (2.9 to 7.9)	1.7 (0.9 to 2.6)
Guatemala	9.4 (6.6 to 12.5)	10.0 (7.4 to 12.9)	6.6 (4.4 to 9.1)	6.6 (3.7 to 10.0)	2.2 (1.2 to 3.3)
Guinea	3.9 (2.7 to 5.3)	4.2 (3.0 to 5.5)	2.7 (1.8 to 3.8)	2.7 (1.5 to 4.1)	0.9 (0.5 to 1.3)
Hungary	9.7 (7.1 to 12.7)	10.4 (7.9 to 13.0)	6.8 (4.7 to 9.2)	6.8 (3.8 to 10.2)	2.3 (1.3 to 3.4)
India	8.7 (5.5 to 12.3)	9.3 (6.1 to 12.8)	6.1 (3.7 to 8.9)	6.1 (3.2 to 9.6)	2.0 (1.0 to 3.1)
Indonesia	6.0 (4.0 to 8.2)	6.4 (4.4 to 8.5)	4.1 (2.6 to 5.9)	4.1 (2.2 to 6.4)	1.3 (0.7 to 2.1)
Iran (Islamic Republic of)	8.5 (6.1 to 11.2)	9.1 (6.8 to 11.6)	5.9 (4.0 to 8.1)	5.9 (3.3 to 8.9)	2.0 (1.1 to 2.9)
Iraq	12.7 (9.6 to 16.2)	13.5 (10.7 to 16.5)	9.0 (6.3 to 12.0)	9.0 (5.3 to 13.2)	3.0 (1.8 to 4.4)
Ireland	8.4 (6.2 to 10.8)	8.9 (6.9 to 11.1)	5.9 (4.0 to 7.9)	5.9 (3.4 to 8.8)	1.9 (1.1 to 2.8)
Italy	10.4 (7.8 to 13.3)	11.1 (8.7 to 13.6)	7.3 (5.1 to 9.7)	7.3 (4.2 to 10.7)	2.4 (1.4 to 3.5)
Jamaica	8.4 (6.1 to 11.1)	8.9 (6.7 to 11.4)	5.8 (3.9 to 8.0)	5.8 (3.3 to 8.7)	1.9 (1.1 to 2.9)
Japan	9.0 (5.1 to 13.5)	9.6 (5.6 to 14.0)	6.3 (3.5 to 9.7)	6.3 (3.0 to 10.4)	2.1 (1.0 to 3.4)
Jordan	3.2 (2.1 to 4.5)	3.5 (2.4 to 4.6)	2.2 (1.4 to 3.2)	2.2 (1.2 to 3.4)	0.7 (0.4 to 1.1)
Kazakhstan	7.2 (4.8 to 9.7)	7.6 (5.3 to 10.1)	5.0 (3.2 to 7.0)	5.0 (2.7 to 7.7)	1.6 (0.9 to 2.5)
Kenya	4.1 (3.1 to 5.3)	4.4 (3.5 to 5.4)	2.8 (2.0 to 3.8)	2.8 (1.6 to 4.2)	0.9 (0.5 to 1.3)
Kiribati	10.2 (7.3 to 13.5)	10.8 (8.1 to 13.8)	7.1 (4.8 to 9.8)	7.1 (4.0 to 10.7)	2.4 (1.3 to 3.5)
Kuwait	15.8 (12.1 to 19.8)	16.7 (13.6 to 20.2)	11.3 (8.0 to 15.0)	11.3 (6.6 to 16.5)	3.9 (2.3 to 5.6)
Kyrgyzstan	3.8 (2.3 to 5.4)	4.0 (2.6 to 5.6)	2.6 (1.5 to 3.8)	2.6 (1.3 to 4.1)	0.8 (0.4 to 1.3)
Lao People's Democratic Republic	4.4 (3.2 to 5.8)	4.7 (3.5 to 6.0)	3.0 (2.0 to 4.1)	3.0 (1.7 to 4.5)	1.0 (0.5 to 1.5)
Latvia	7.6 (5.4 to 10.1)	8.1 (6.0 to 10.5)	5.3 (3.5 to 7.3)	5.3 (3.0 to 8.0)	1.7 (1.0 to 2.6)
Lebanon	9.3 (6.6 to 12.3)	9.8 (7.4 to 12.6)	6.5 (4.4 to 8.8)	6.5 (3.6 to 9.8)	2.1 (1.2 to 3.2)
Lesotho	1.7 (1.2 to 2.4)	1.9 (1.3 to 2.5)	1.2 (0.8 to 1.7)	1.2 (0.6 to 1.8)	0.4 (0.2 to 0.6)

Liberia	6.6 (4.7 to 8.7)	7.0 (5.3 to 8.9)	4.6 (3.1 to 6.2)	4.6 (2.6 to 6.8)	1.5 (0.8 to 2.2)
Libya	9.3 (6.6 to 12.4)	9.8 (7.3 to 12.7)	6.5 (4.3 to 8.9)	6.5 (3.7 to 9.9)	2.2 (1.2 to 3.2)
Lithuania	6.9 (4.9 to 9.2)	7.4 (5.5 to 9.5)	4.8 (3.2 to 6.6)	4.8 (2.7 to 7.2)	1.6 (0.9 to 2.3)
Luxembourg	7.4 (5.4 to 9.6)	7.9 (6.0 to 9.9)	5.1 (3.5 to 7.0)	5.1 (2.9 to 7.6)	1.7 (1.0 to 2.5)
Madagascar	4.6 (3.3 to 6.1)	4.9 (3.7 to 6.3)	3.2 (2.1 to 4.4)	3.2 (1.8 to 4.7)	1.0 (0.6 to 1.5)
Malawi	4.2 (3.2 to 5.3)	4.5 (3.6 to 5.5)	2.9 (2.0 to 3.8)	2.9 (1.7 to 4.2)	0.9 (0.5 to 1.4)
Malaysia	9.8 (7.0 to 12.9)	10.4 (7.8 to 13.4)	6.9 (4.6 to 9.4)	6.9 (3.9 to 10.4)	2.3 (1.3 to 3.4)
Maldives	7.8 (5.4 to 10.7)	8.3 (5.9 to 11.0)	5.4 (3.5 to 7.7)	5.4 (3.0 to 8.4)	1.8 (1.0 to 2.7)
Mali	10.2 (7.6 to 13.0)	10.8 (8.5 to 13.4)	7.1 (4.9 to 9.5)	7.1 (4.2 to 10.5)	2.4 (1.4 to 3.5)
Malta	10.5 (7.7 to 13.5)	11.1 (8.6 to 13.9)	7.3 (5.0 to 9.9)	7.3 (4.2 to 10.9)	2.4 (1.4 to 3.6)
Marshall Islands	10.9 (7.7 to 14.3)	11.5 (8.7 to 14.7)	7.6 (5.2 to 10.4)	7.6 (4.3 to 11.4	2.5 (1.4 to 3.8)
Mauritania	10.4 (7.7 to 13.4)	11.0 (8.6 to 13.7)	7.3 (5.0 to 9.8)	7.3 (4.2 to 10.7)	2.4 (1.4 to 3.5)
Mauritius	7.7 (5.4 to 10.2)	8.2 (6.1 to 10.5)	5.4 (3.6 to 7.4)	5.4 (3.0 to 8.1)	1.8 (1.0 to 2.7)
Mexico	7.5 (5.4 to 9.8)	8.0 (6.1 to 10.1)	5.2 (3.6 to 7.1)	5.2 (3.0 to 7.9)	1.7 (1.0 to 2.5)
Micronesia (Federated States of)	9.3 (6.7 to 12.3)	9.9 (7.3 to 12.7)	6.5 (4.4 to 9.0)	6.5 (3.7 to 9.8)	2.2 (1.2 to 3.2)
Mongolia	5.0 (3.5 to 6.7)	5.3 (3.8 to 6.9)	3.4 (2.3 to 4.8)	3.4 (1.9 to 5.2)	1.1 (0.6 to 1.7)
Morocco	6.8 (4.6 to 9.4)	7.3 (5.1 to 9.7)	4.7 (3.0 to 6.7)	4.7 (2.5 to 7.3)	1.6 (0.8 to 2.4)
Mozambique	1.5 (1.0 to 2.1)	1.7 (1.2 to 2.2)	1.1 (0.7 to 1.59)	1.1 (0.6 to 1.6)	0.3 (0.2 to 0.5)
Myanmar	2.9 (1.9 to 4.1)	3.1 (2.1 to 4.2)	2.0 (1.2 to 2.9)	2.0 (1.1 to 3.1)	0.6 (0.3 to 1.0)
Namibia	8.6 (6.2 to 11.2)	9.1 (6.9 to 11.5)	6.0 (4.1 to 8.1)	6.0 (3.4 to 8.9)	2.0 (1.1 to 2.9)
Nauru	10.5 (7.4 to 14.1)	11.2 (8.3 to 14.6)	7.4 (4.9 to 10.2)	7.4 (4.2 to 11.2)	2.5 (1.4 to 3.7)
Nepal	3.6 (2.7 to 4.6)	3.9 (3.1 to 4.8)	2.5 (1.7 to 3.3)	2.5 (1.4 to 3.6)	0.8 (0.5 to 1.2)
Netherlands	7.1 (5.2 to 9.3)	7.5 (5.7 to 9.5)	4.9 (3.3 to 6.7)	4.9 (2.8 to 7.3)	1.6 (0.9 to 2.4)
New Zealand	10.6 (8.0 to 13.7)	11.3 (8.9 to 13.9)	7.5 (5.2 to 10.0)	7.5 (4.3 to 11.0)	2.5 (1.4 to 3.6)
Niger	5.9 (4.1 to 8.0)	6.3 (4.5 to 8.3)	4.1 (2.6 to 5.7)	4.1 (2.3 to 6.2)	1.3 (0.7 to 2.0)
Nigeria	7.1 (5.1 to 9.2)	7.5 (5.7 to 9.5)	4.9 (3.3 to 6.7)	4.9 (2.8 to 7.4)	1.6 (0.9 to 2.4)
Niue	1.9 (1.2 to 2.7)	2.0 (1.4 to 2.8)	1.3 (0.8 to 1.9)	1.3 (0.7 to 2.1)	0.4 (0.2 to 0.7)
Norway	8.2 (6.0 to 10.6)	8.7 (6.7 to 10.9)	5.7 (3.9 to 7.7)	5.7 (3.2 to 8.4)	1.9 (1.1 to 2.7)
Oman	8.4 (6.0 to 11.3)	9.0 (6.7 to 11.6)	5.9 (3.9 to 8.1)	5.9 (3.3 to 8.9)	1.9 (1.1 to 2.9)
Pakistan	8.6 (5.3 to 12.5)	9.2 (5.9 to 12.9)	6.0 (3.6 to 8.9)	6.0 (3.1 to 9.7)	2.0 (1.0 to 3.2)
Palau	10.3 (7.3 to 13.6)	10.9 (8.2 to 14.1)	7.2 (4.8 to 9.9)	7.2 (4.1 to 10.8)	2.4 (1.3 to 3.6)
Papua New Guinea	4.0 (2.4 to 5.7)	4.3 (2.7 to 6.0)	2.7 (1.6 to 4.1)	2.7 (1.4 to 4.4)	0.9 (0.4 to 1.4)
Paraguay	9.5 (6.8 to 12.5)	10.1 (7.6 to 12.8)	6.6 (4.5 to 9.1)	6.6 (3.7 to 10.0)	2.2 (1.2 to 3.3)
Philippines	10,0 (7.3 to 13.1)	10.6 (8.1 to 13.4)	7.0 (4.8 to 9.6)	7.0 (3.9 to 10.5)	2.3 (1.3 to 3.4)
Poland	8.3 (6.0 to 11.1)	8.9 (6.7 to 11.4)	5.8 (3.9 to 8.0)	5.8 (3.3 to 8.7)	1.9 (1.1 to 2.8)
Portugal	10.8 (7.9 to 14.0)	11.5 (9.0 to 14.4)	7.6 (5.3 to 10.3)	7.6 (4.4 to 11.3)	2.5 (1.5 to 3.7)
Qatar	9.3 (6.6 to 12.3)	9.9 (7.4 to 12.7)	6.5 (4.4 to 9.0)	6.5 (3.7 to 9.9)	2.2 (1.2 to 3.2)
Republic of Korea	9.0 (5.3 to 13.3)	9.6 (5.8 to 13.7)	6.3 (3.5 to 9.5)	6.3 (3.1 to 10.3)	2.1 (1.0 to 3.4)
Republic of Moldova	3.1 (2.1to 4.3)	3.3 (2.3 to 4.5)	2.1 (1.3 to 3.2)	2.1 (1.1 to 3.3)	0.7 (0.4 to 1.1)
Romania	9.0 (6.5 to 11.8)	9.6 (7.3 to 12.1)	6.3 (4.3 to 8.7)	6.3 (3.6 to 9.4)	2.1 (1.2 to 3.1)
Russian Federation	4.6 (3.2 to 6.1)	4.9 (3.5 to 6.4)	3.2 (2.1 to 4.4)	3.2 (1.8 to 4.8)	1.0 (0.6 to 1.5)
Rwanda	3.9 (2.7 to 5.3)	4.2 (3.1 to 5.45)	2.7 (1.8 to 3.8)	2.7 (1.5 to 4.1)	0.9 (0.5 to 1.3)
Saint Kitts & Nevis	8.3 (5.8, 10.9)	8.8 (6.5 to 11.4)	5.8 (3.8 to 8.0)	5.8 (3.2 to 8.7)	1.9 (1.1 to 2.8)

Saint Lucia	10.0 (7.1 to 13.2)	10.7 (7.9 to 13.7)	7.0 (4.7 to 9.7)	7.0 (4.0 to 10.6)	2.3 (1.3 to 3.5)
Samoa	3.4 (2.2 to 4.8)	3.6 (2.5 to 5.0)	2.3 (1.4 to 3.4)	2.3 (1.3 to 3.7)	0.8 (0.4 to 1.2)
Sao Tome & Principe	4.2 (2.9 to 5.6)	4.4 (3.2 to 5.8)	2.9 (1.9 to 4.0)	2.9 (1.6 to 4.4)	0.9 (0.5 to 1.4)
Saudi Arabia	12.9 (9.7 to 16.6)	13.7 (10.9 to 16.9)	9.2 (6.4 to 12.2)	9.2 (5.3 to 13.5)	3.1 (1.8 to 4.5)
Senegal	6.1 (4.5 to 7.8)	6.5 (5.0 to 8.1)	4.2 (2.9 to 5.6)	4.2 (2.4 to 6.2)	1.4 (0.8 to 2.0)
Serbia	10.0 (7.1 to 13.1)	10.6 (8.1 to 13.5)	7.0 (4.7 to 9.5)	7.1 (3.9 to 10.5)	2.3 (1.3 to 3.4)
Seychelles	5.0 (3.4 to 6.9)	5.3 (3.8 to 7.0)	3.5 (2.2 to 4.9)	3.5 (1.9 to 5.3)	1.1 (0.6 to 1.7)
Sierra Leone	3.9 (2.7 to 5.2)	4.1 (3.0 to 5.3)	2.7 (1.7 to 3.7)	2.7 (1.5 to 4.0)	0.9 (0.5 to 1.3)
Singapore	9.3 (5.5 to 13.5)	9.9 (6.0 to 14.1)	6.5 (3.6 to 9.8)	6.5 (3.1 to 10.6)	2.1 (1.1 to 3.5)
Slovakia	8.9 (6.5 to 11.7)	9.5 (7.2 to 11.9)	6.2 (4.2 to 8.5)	6.2 (3.5 to 9.3)	2.1 (1.2 to 3.0)
Slovenia	8.3 (5.9 to 10.9)	8.8 (6.7 to 11.2)	5.8 (3.9 to 7.8)	5.8 (3.3 to 8.7)	1.9 (1.1 to 2.8)
Solomon Islands	4.9 (3.2 to 6.7)	5.2 (3.6 to 6.9)	3.3 (2.1 to 4.8)	3.3 (1.8 to 5.2)	1.1 (0.6 to 1.7)
South Africa	9.7 (7.1 to 12.7)	10.3 (7.8 to 12.9)	6.8 (4.6 to 9.2)	6.8 (3.9 to 10.1)	2.2 (1.3 to 3.3)
Spain	7.0 (5.1 to 9.2)	7.4 (5.7 to 9.4)	4.9 (3.3 to 6.6)	4.9 (2.7 to 7.3)	1.6 (0.9 to 2.4)
Sri Lanka	7.5 (5.2 to 10.2)	8.0 (5.7 to 10.5)	5.2 (3.4 to 7.3)	5.2 (2.9 to 7.9)	1.7 (0.9 to 2.6)
State of Palestine	10.2 (7.4 to 13.2)	10.8 (8.3 to 13.6)	7.2 (4.9 to 9.7)	7.2 (4.1 to 10.6)	2.4 (1.4 to 3.5)
Suriname	11.1 (8.2 to 14.3)	11.8 (9.2 to 14.6)	7.8 (5.4 to 10.5)	7.8 (4.5 to 11.5)	2.6 (1.5 to 3.8)
Swaziland	7.3 (5.4 to 9.4)	7.8 (6.1 to 9.6)	5.1 (3.5 to 6.8)	5.1 (2.9 to 7.4)	1.7 (0.9 to 2.4)
Sweden	6.1 (4.4 to 8.0)	6.5 (4.9 to 8.3)	4.2 (2.9 to 5.8)	4.2 (2.4 to 6.3)	1.4 (0.8 to 2.0)
Switzerland	6.2 (4.4 to 8.3)	6.6 (4.9 to 8.5)	4.3 (2.9 to 6.0)	4.3 (2.4 to 6.6)	1.4 (0.8 to 2.1)
Tajikistan	7.6 (5.2 to 10.3)	8.1 (5.8 to 10.6)	5.3 (3.4 to 7.4)	5.3 (2.9 to 8.1)	1.7 (0.9 to 2.6)
Thailand	6.4 (4.5 to 8.7)	6.8 (5.0 to 9.0)	4.5 (2.9 to 6.3)	4.5 (2.5 to 6.9)	1.5 (0.8 to 2.2)
Timor-Leste	4.8 (3.1 to 6.6)	5.1 (3.4 to 6.9)	3.3 (2.0 to 4.8)	3.3 (1.7 to 5.1)	1.1 (0.5 to 1.7)
Togo	2.7 (1.9 to 3.6)	2.9 (2.1 to 3.7)	1.8 (1.2 to 2.6)	1.8 (1.0 to 2.8)	0.6 (0.3 to 0.9)
Tokelau	3.0 (1.9 to 4.3)	3.2 (2.1 to 4.4)	2.1 (1.2 to 3.1)	2.1 (1.1 to 3.3)	0.7 (0.3 to 1.1)
Tonga	4.7 (3.1 to 6.4)	5.0 (3.4 to 6.6)	3.2 (2.0 to 4.6)	3.2 (1.7 to 4.9)	1.0 (0.6 to 1.6)
Trinidad and Tobago	9.7 (6.7 to 13.1)	10.3 (7.4 to 13.5)	6.8 (4.4 to 9.5)	6.8 (3.7 to 10.3)	2.2 (1.2 to 3.4)
Tunisia	7.8 (5.6 to 10.5)	8.4 (6.2 to 10.7)	5.5 (3.6 to 7.6)	5.5 (3.1 to 8.3)	1.8 (1.0 to 2.7)
Turkey	7.9 (5.6 to 10.5)	8.4 (6.3 to 10.9)	5.5 (3.7 to 7.6)	5.5 (3.1 to 8.3)	1.8 (1.0 to 2.7)
Tuvalu	7.1 (4.9 to 9.5)	7.6 (5.5 to 9.9)	4.9 (3.3 to 6.9)	4.9 (2.7 to 7.6)	1.6 (0.9 to 2.4)
Uganda	1.5 (1.0 to 2.1)	1.6 (1.1 to 2.2)	1.0 (0.7 to 1.5)	1.0 (0.6 to 1.6)	0.3 (0.2 to 0.5)
Ukraine	5.2 (3.5 to 7.2)	5.6 (3.9 to 7.4)	3.6 (2.3 to 5.1)	3.6 (2.0 to 5.6)	1.2 (0.6 to 1.8)
United Arab Emirates	10.4 (7.6 to 13.6)	11.1 (8.5 to 13.9)	7.3 (5.0 to 9.9)	7.3 (4.1 to 10.9)	2.4 (1.4 to 3.6)
United Kingdom	9.1 (6.8 to 11.8)	9.7 (7.6 to 12.1)	6.4 (4.4 to 8.6)	6.4 (3.7 to 9.4)	2.1 (1.2 to 3.1)
United Republic of Tanzania	1.8 (1.2 to 2.4)	1.9 (1.4 to 2.5)	1.2 (0.8 to 1.7)	1.2 (0.7 to 1.9)	0.4 (0.2 to 0.6)
United States of America	10.1 (7.5 to 12.9)	10.7 (8.3 to 13.2)	7.1 (4.9 to 9.5)	7.1 (4.0 to 10.6)	2.3 (1.3 to 3.4)
Uruguay	5.9 (4.2 to 7.9)	6.3 (4.6 to 8.1)	4.1 (2.7 to 5.7)	4.1 (2.3 to 6.2)	1.3 (0.7 to 2.0)
Uzbekistan	5.1 (3.3 to 7.1)	5.4 (3.7 to 7.3)	3.5 (2.2 to 5.0)	3.5 (1.8 to 5.5)	1.1 (0.6 to 1.8)
Vanuatu	2.2 (1.4 to 3.1)	2.3 (1.5 to 3.2)	1.5 (0.9 to 2.2)	1.5 (0.8 to 2.4)	0.5 (0.3 to 0.8)
Venezuela (Bolivarian Republic of)	8.1 (5.8 to 10.8)	8.6 (6.3 to 11.2)	5.6 (3.7 to 7.8)	5.6 (3.1 to 8.5)	1.9 (1.0 to 2.8)
Viet Nam	6.6 (4.6 to 9.0)	7.1 (5.2 to 9.3)	4.6 (3.0 to 6.5)	4.6 (2.6 to 7.0)	1.5 (0.8 to 2.3)
Zambia	5.8 (4.3 to 7.6)	6.2 (4.8 to 7.8)	4.0 (2.8 to 5.5)	4.0 (2.3 to 6.0)	1.3 (0.7 to 1.9)
Zimbabwe	7.0 (4.9 to 9.3)	7.4 (5.5 to 9.6)	4.9 (3.2 to 6.8)	4.9 (2.7 to 7.4)	1.6 (0.9 to 2.4)

Supplementary Table 2. Population Attributable Risks for Physical Inactivity and General Cancer Outcomes by Country

Country	Bladder Cancer	Colon Cancer	Esophageal Cancer	Gastric Cancer	Renal Cancer
Algeria	2.6 (-1.6 to 7.5)	3.6 (1.4 to 6.0)	8.6 (-3.1 to 25.7)	8.3 (-0.2 to 20.1)	8.6 (2.8 to 15.7)
American Samoa	4.1 (-2.3 to 11.4)	5.6 (2.2 to 9.3)	13.0 (-4.5 to 39.0)	12.6 (-0.6 to 30.4)	13.0 (4.2 to 23.6)
Andorra	3.0 (-1.7 to 8.4)	4.1 (1.6 to 6.7)	9.7 (-3.5 to 29.1)	9.4 (-0.4 to 22.8)	9.7 (3.1 to 17.7)
Argentina	3.2 (-1.8 to 9.0)	4.4 (1.7 to 7.3)	10.4 (-3.6 to 31.1)	10.1 (-0.2 to 24.0)	10.4 (3.5 to 19.1)
Armenia	1.8 (-1.0 to 5.1)	2.4 (0.9 to 4.2)	6.0 (-2.0 to 17.9)	5.8 (-0.2 to 13.9)	6.0 (1.9 to 11.1)
Australia	2.4 (-1.4 to 6.8)	3.2 (1.3 to 5.5)	7.8 (-2.9 to 23.2)	7.6 (-0.3 to 18.1)	7.8 (2.7 to 14.3)
Austria	2.4 (-1.4 to 6.6)	3.2 (1.3 to 5.4)	7.8 (-2.8 to 22.7)	7.5 (-0.3 to 17.9)	7.8 (2.5 to 14.2)
Bahamas	3.4 (-1.8 to 9.5)	4.6 (1.8 to 7.6)	10.8 (-3.7 to 32.2)	10.5 (-0.5 to 24.9)	10.8 (3.5 to 19.8)
Bangladesh	2.2 (-1.3 to 6.3)	3.0 (1.1 to 5.3)	7.2 (-2.5 to 22.0)	7.0 (-0.3 to 17.4)	7.2 (2.3 to 13.8)
Barbados	3.3 (-1.9 to 9.3)	4.5 (1.8 to 7.6)	10.7 (-3.6 to 31.5)	10.4 (-0.5 to 25.2)	10.7 (3.5 to 19.5)
Belarus	1.1 (-0.7 to 3.3)	1.5 (0.6 to 2.7)	3.8 (-1.2 to 11.3)	3.7 (-0.2 to 9.0)	3.8 (1.2 to 7.1)
Belgium	2.8 (-1.6 to 7.8)	3.8 (1.5 to 6.4)	9.1 (-3.4 to 27.1)	8.8 (-0.4 to 21.3)	9.1 (2.9 to 16.5)
Benin	1.3 (-0.7 to 3.6)	1.7 (0.7 to 3.0)	4.3 (-1.5 to 12.8)	4.1 (-0.1 to 9.7)	4.3 (1.4 to 7.9)
Bermuda	2.1 (-1.2 to 5.9)	2.8 (1.1 to 4.9)	6.8 (-2.4 to 20.5)	6.6 (-0.3 to 15.9)	6.8 (2.2 to 12.7)
Bhutan	1.8 (-1.1 to 5.1)	2.5 (1.0 to 4.2)	6.1 (-2.1 to 17.9)	5.9 (-0.3 to 13.9)	6.1 (2.0 to 11.0)
Bosnia & Herzegovina	2.0 (-1.1 to 5.7)	2.7 (1.1 to 4.7)	6.7 (-2.3 to 20.0)	6.4 (-0.3 to 15.7)	6.7 (2.2 to 12.4)
Botswana	1.7 (-0.9 to 4.8)	2.3 (0.9 to 3.9)	5.7 (-2.1 to 17.2)	5.5 (-0.2 to 13.2)	5.7 (1.9 to 10.4)
Brazil	3.6 (-2.2 to 10.1)	4.9 (2.0 to 8.2)	11.6 (-3.7 to 34.6)	11.3 (-0.6 to 27.2)	11.6 (3.9 to 21.1)
British Virgin Islands	2.1 (-1.2 to 6.1)	2.9 (1.2 to 5.0)	7.1 (-2.4 to 21.3)	6.9 (-0.3 to 16.8)	7.1 (2.3 to 13.0)
Brunei Darussalam	2.1 (-1.2 to 6.3)	2.9 (1.1 to 5.1)	7.1 (-2.5 to 21.4)	6.9 (-0.3 to 16.8)	7.1 (2.2 to 13.5)
Bulgaria	3.0 (-1.7 to 8.6)	4.1 (1.6 to 6.9)	9.8 (-3.3 to 29.0)	9.4 (-0.5 to 22.6)	9.8 (3.2 to 17.7)
Burkina Faso	1.6 (-0.9 to 4.6)	2.2 (0.9 to 3.7)	5.4 (-1.9 to 15.9)	5.2 (-0.3 to 12.4)	5.4 (1.7 to 9.9)
Cabo Verde	1.6 (-0.9 to 4.4)	2.1 (0.8 to 3.6)	5.2 (-1.7 to 15.6)	5.1 (-0.2 to 11.9)	5.2 (1.7 to 9.6)
Cambodia	0.8 (-0.5 to 2.4)	1.1 (0.4 to 2.0)	2.9 (-1.0 to 8.7)	2.8 (-0.1 to 6.8)	2.9 (0.9 to 5.5)
Cameroon	2.2 (-1.2 to 6.3)	3.0 (1.2 to 5.1)	7.4 (-2.5 to 21.7)	7.2 (-0.3 to 17.0)	7.4 (2.5 to 13.5)
Canada	2.2 (-1.2 to 6.3)	3.1 (1.2 to 5.1)	7.4 (-2.6 to 22.0)	7.2 (-0.3 to 17.0)	7.4 (2.4 to 13.8)
Cayman Islands	2.3 (-1.3 to 6.4)	3.1 (1.2 to 5.4)	7.5 (-2.6 to 22.7)	7.3 (-0.6 to 17.5)	7.5 (2.6 to 13.9)
Central African Republic	1.1 (-0.6 to 3.2)	1.6 (0.6 to 2.6)	3.9 (-1.3 to 11.7)	3.7 (-0.2 to 8.9)	3.9 (1.2 to 7.07)
Chad	1.8 (-1.1 to 5.3)	2.5 (1.0 to 4.3)	6.1 (-2.2 to 18.2)	5.9 (-0.4 to 14.2)	6.1 (2.0 to 11.38)
Chile	2.1 (-1.3 to 5.9)	2.8 (1.1 to 4.9)	6.9 (-2.4 to 20.3)	6.7 (-0.3 to 16.1)	6.9 (2.3 to 12.76)
China	1.1 (-0.7 to 3.2)	1.5 (0.6 to 2.6)	3.8 (-1.2 to 11.4)	3.7 (-0.2 to 8.9)	3.8 (1.2 to 7.15)
Colombia	3.4 (-2.0 to 9.7)	4.6 (1.9 to 7.7)	11.0 (-3.6 to 32.5)	10.6 (-0.3 to 25.4)	11.0 (3.6 to 19.7)
Comoros	1.1 (-0.7 to 3.2)	1.6 (0.6 to 2.6)	3.9 (-1.3 to 11.3)	3.7 (-0.2 to 8.9)	3.9 (1.3 to 7.1)
Congo	2.2 (-1.2 to 6.3)	3.0 (1.2 to 5.0)	7.3 (-2.6 to 21.7)	7.0 (-0.3 to 16.8)	7.3 (2.4 to 13.3)
Cook Islands	1.5 (-0.8 to 4.2)	2.0 (0.8 to 3.4)	4.9 (-1.7 to 14.7)	4.8 (-0.3 to 11.5)	4.9 (1.7 to 9.1)
Costa Rica	3.6 (-2.1 to 10.2)	4.8 (1.8 to 8.1)	11.4 (-4.2 to 34.1)	11.1 (-0.5 to 26.0)	11.4 (3.7 to 20.6)
Cote d'Ivoire	2.6 (-1.5 to 7.3)	3.5 (1.4 to 5.9)	8.5 (-3.0 to 24.8)	8.2 (-0.3 to 19.4)	8.5 (2.6 to 15.5)
Croatia	2.4 (-1.3 to 6.8)	3.3 (1.3 to 5.6)	8.0 (-3.0 to 23.9)	7.8 (-0.2 to 18.4)	8.0 (2.6 to 14.7)

Cuba	2.9 (-1.7 to 8.1)	3.9 (1.6 to 6.6)	9.4 (-3.2 to 27.6)	9.1 (-0.5 to 21.8)	9.4 (3.2 to 17.1)
Cyprus	3.4 (-1.9 to 9.9)	4.7 (1.8 to 7.7)	11.1 (-4.1 to 32.9)	10.7 (-0.3 to 25.9)	11.1 (3.8 to 20.0)
Czech Republic	2.4 (-1.4 to 6.9)	3.3 (1.3 to 5.6)	8.0 (-2.6 to 24.1)	7.8 (-0.3 to 18.7)	8.0 (2.5 to 14.7)
Democratic Republic of the Congo	1.9 (-1.1 to 5.4)	2.6 (1.0 to 4.3)	6.3 (-2.2 to 18.5)	6.0 (-0.2 to 14.4)	6.3 (2.1 to 11.5)
Denmark	2.2 (-1.3 to 6.3)	3.0 (1.2 to 5.1)	7.4 (-2.6 to 21.9)	7.2 (-0.3 to 16.9)	7.4 (2.4 to 13.6)
Dominica	1.7 (-0.9 to 4.9)	2.3 (0.9 to 4.0)	5.7 (-2.0 to 17.0)	5.5 (-0.3 to 13.2)	5.7 (2.0 to 10.5)
Dominican Republic	3.0 (-1.7 to 8.6)	4.1 (1.6 to 7.0)	9.8 (-3.4 to 29.1)	9.5 (-0.3 to 22.7)	9.8 (3.2 to 17.8)
Ecuador	2.1 (-1.2 to 5.9)	2.9 (1.1 to 5.0)	7.1 (-2.5 to 21.5)	6.8 (-0.2 to 16.4)	7.1 (2.3 to 13.0)
Egypt	2.4 (-1.3 to 6.8)	3.3 (1.3 to 5.6)	8.0 (-2.9 to 23.6)	7.7 (-0.3 to 18.6)	8.0 (2.6 to 14.7)
Eritrea	1.8 (-1.0 to 4.9)	2.4 (1.0 to 4.0)	5.9 (-2.2 to 17.2)	5.7 (-0.2 to 13.5)	5.9 (2.0 to 10.7)
Estonia	2.5 (-1.4 to 7.0)	3.4 (1.3 to 5.7)	8.2 (-2.7 to 24.4)	8.0 (-0.3 to 19.0)	8.2 (2.7 to 15.2)
Ethiopia	1.2 (-0.7 to 3.3)	1.6 (0.7 to 2.7)	4.0 (-1.3 to 11.8)	3.9 (-0.2 to 9.1)	4.0 (1.3 to 7.3)
Fiji	1.4 (-0.8 to 3.9)	1.9 (0.7 to 3.3)	4.7 (-1.5 to 13.7)	4.5 (-0.2 to 10.7)	4.7 (1.5 to 8.6)
Finland	1.3 (-0.8 to 3.8)	1.8 (0.7 to 3.0)	4.4 (-1.4 to 13.3)	4.3 (-0.2 to 10.3)	4.4 (1.5 to 8.1)
France	2.3 (-1.3 to 6.5)	3.1 (1.2 to 5.2)	7.6 (-2.6 to 22.7)	7.3 (-0.4 to 17.5)	7.6 (2.4 to 13.7)
French Polynesia	1.4 (-0.8 to 4.1)	1.9 (0.7 to 3.4)	4.8 (-1.7 to 14.1)	4.6 (-0.3 to 11.1)	4.8 (1.5 to 8.8)
Gabon	2.0 (-1.1 to 5.6)	2.7 (1.1 to 4.7)	6.6 (-2.4 to 19.7)	6.4 (-0.1 to 15.5)	6.6 (2.0 to 12.3)
Gambia	1.7 (-1.0 to 4.8)	2.3 (0.9 to 3.9)	5.6 (-1.8 to 16.5)	5.4 (-0.3 to 12.8)	5.6 (1.9 to 10.2)
Georgia	1.4 (-0.8 to 4.1)	1.9 (0.8 to 3.4)	4.8 (-1.6 to 14.3)	4.6 (-0.2 to 11.3)	4.8 (1.5 to 9.0)
Germany	3.3 (-1.9 to 9.1)	4.4 (1.8 to 7.3)	10.6 (-3.8 to 31.3)	10.2 (-0.6 to 24.4)	10.6 (3.4 to 19.1)
Ghana	1.7 (-1.1 to 4.8)	2.3 (0.9 to 3.9)	5.8 (-1.9 to 16.9)	5.6 (-0.1 to 13.1)	5.8 (1.9 to 10.4)
Greece	2.9 (-1.8 to 8.4)	4.0 (1.6 to 6.6)	9.6 (-3.5 to 28.3)	9.2 (-0.6 to 22.0)	9.6 (3.2 to 17.2)
Grenada	2.2 (-1.3 to 6.4)	3.1 (1.2 to 5.2)	7.4 (-2.6 to 22.5)	7.2 (-0.3 to 17.5)	7.4 (2.4 to 13.9)
Guatemala	2.9 (-1.7 to 8.1)	3.9 (1.6 to 6.7)	9.4 (-3.3 to 28.4)	9.1 (-0.4 to 21.9)	9.4 (3.1 to 17.5)
Guinea	1.2 (-0.7 to 3.3)	1.6 (0.6 to 2.7)	3.9 (-1.4 to 11.3)	3.8 (-0.1 to 8.9)	3.9 (1.2 to 7.3)
Hungary	3.0 (-1.7 to 8.4)	4.1 (1.6 to 6.8)	9.7 (-3.4 to 29.0)	9.4 (-0.3 to 22.2)	9.7 (3.2 to 17.5)
India	2.7 (-1.6 to 7.7)	3.6 (1.3 to 6.3)	8.7 (-2.9 to 26.8)	8.4 (-0.3 to 20.5)	8.7 (2.7 to 16.6)
Indonesia	1.8 (-0.9 to 5.1)	2.4 (0.9 to 4.2)	6.0 (-2.1 to 17.7)	5.8 (-0.3 to 13.8)	6.0 (1.9 to 11.1)
Iran (Islamic Republic of)	2.6 (-1.4 to 7.3)	3.5 (1.4 to 6.0)	8.5 (-2.9 to 25.2)	8.2 (-0.6 to 19.6)	8.5 (2.8 to 15.7)
Iraq	4.0 (-2.2 to 11.2)	5.4 (2.2 to 9.0)	12.7 (-4.1 to 38.2)	12.3 (-0.8 to 29.1)	12.7 (4.3 to 23.0)
Ireland	2.6 (-1.5 to 7.2)	3.5 (1.4 to 5.8)	8.4 (-2.8 to 24.8)	8.1 (-0.1 to 19.3)	8.4 (2.7 to 15.1)
Italy	3.2 (-1.7 to 9.2)	4.4 (1.7 to 7.2)	10.4 (-3.7 to 31.1)	10.1 (-0.4 to 23.8)	10.4 (3.5 to 18.9)
Jamaica	2.5 (-1.5 to 7.1)	3.5 (1.4 to 5.8)	8.4 (-2.9 to 25.0)	8.1 (-0.3 to 19.4)	8.4 (2.7 to 15.3)
Japan	2.8 (-1.6 to 8.0)	3.8 (1.3 to 6.9)	9.0 (-2.9 to 27.4)	8.8 (-0.2 to 22.3)	9.0 (2.7 to 17.6)
Jordan	0.9 (-0.5 to 2.7)	1.3 (0.5 to 2.3)	3.2 (-1.2 to 9.7)	3.1 (-0.1 to 7.5)	3.2 (1.0 to 6.0)
Kazakhstan	2.2 (-1.2 to 6.1)	2.9 (1.1 to 5.0)	7.2 (-2.3 to 21.2)	6.9 (-0.2 to 16.8)	7.2 (2.3 to 13.4)
Kenya	1.2 (-0.7 to 3.4)	1.7 (0.7 to 2.8)	4.1 (-1.4 to 12.2)	4.0 (-0.2 to 9.4)	4.1 (1.4 to 7.5)
Kiribati	3.1 (-1.9 to 9.0)	4.3 (1.7 to 7.2)	10.2 (-3.3 to 31.1)	9.8 (-0.2 to 23.7)	10.2 (3.2 to 18.5)
Kuwait	5.1 (-3.0 to 14.3)	6.9 (2.9 to 11.4)	15.8 (-5.4 to 47.9)	15.3 (-0.5 to 37.0)	15.8 (5.3 to 28.8)
Kyrgyzstan	1.1 (-0.6 to 3.2)	1.5 (0.6 to 2.7)	3.8 (-1.3 to 11.3)	3.6 (-0.2 to 8.8)	3.8 (1.2 to 7.1)
Lao People's Democratic Republic	1.3 (-0.8 to 3.6)	1.8 (0.7 to 3.0)	4.4 (-1.5 to 13.0)	4.2 (-0.2 to 10.2)	4.4 (1.4 to 7.9)
Latvia	2.3 (-1.3 to 6.5)	3.1 (1.2 to 5.3)	7.6 (-2.6 to 23.0)	7.4 (-0.2 to 17.5)	7.6 (2.6 to 13.9)
Lebanon	2.8 (-1.5 to 8.1)	3.9 (1.5 to 6.5)	9.3 (-3.1 to 27.7)	9.0 (-0.4 to 21.3)	9.3 (3.0 to 16.9)

Lesotho	0.5 (-0.3 to 1.4)	0.7 (0.3 to 1.2)	1.7 (-0.6 to 5.2)	1.7 (-0.1 to 4.0)	1.7 (0.6 to 3.2)
Liberia	2.0 (-1.1 to 5.5)	2.7 (1.1 to 4.5)	6.6 (-2.3 to 19.3)	6.4 (-0.3 to 15.2)	6.6 (2.1 to 12.0)
Libya	2.8 (-1.7 to 8.1)	3.9 (1.5 to 6.5)	9.3 (-3.2 to 27.5)	9.0 (-0.4 to 21.6)	9.3 (2.9 to 17.1)
Lithuania	2.1 (-1.2 to 5.8)	2.8 (1.1 to 4.8)	6.9 (-2.3 to 19.9)	6.7 (-0.3 to 15.9)	6.9 (2.3 to 12.7)
Luxembourg	2.2 (-1.2 to 6.2)	3.0 (1.2 to 5.1)	7.4 (-2.6 to 21.5)	7.1 (-0.3 to 16.9)	7.4 (2.4 to 13.3)
Madagascar	1.4 (-0.8 to 3.8)	1.9 (0.7 to 3.1)	4.6 (-1.5 to 13.7)	4.4 (-0.2 to 10.6)	4.6 (1.5 to 8.4)
Malawi	1.2 (-0.7 to 3.4)	1.7 (0.7 to 2.8)	4.2 (-1.5 to 12.2)	4.0 (-0.1 to 9.6)	4.2 (1.4 to 7.6)
Malaysia	3.0 (-1.7 to 8.6)	4.1 (1.6 to 6.9)	9.8 (-3.3 to 29.2)	9.5 (-0.4 to 22.5)	9.8 (3.3 to 18.2)
Maldives	2.4 (-1.3 to 6.8)	3.2 (1.3 to 5.5)	7.8 (-2.6 to 23.6)	7.6 (-0.3 to 18.2)	7.8 (2.6 to 14.5)
Mali	3.1 (-1.8 to 8.8)	4.3 (1.7 to 7.0)	10.2 (-3.4 to 30.0)	9.8 (-0.2 to 23.4)	10.2 (3.3 to 18.6)
Malta	3.2 (-1.9 to 9.0)	4.4 (1.8 to 7.3)	10.5 (-3.7 to 30.7)	10.1 (-0.3 to 24.1)	10.5 (3.4 to 19.2)
Marshall Islands	3.4 (-1.9 to 9.5)	4.6 (1.8 to 7.8)	10.9 (-3.5 to 32.6)	10.5 (-0.2 to 25.3)	10.9 (3.6 to 20.0)
Mauritania	3.2 (-1.9 to 8.9)	4.4 (1.8 to 7.2)	10.4 (-3.6 to 31.1)	10.0 (-0.6 to 23.8)	10.4 (3.5 to 18.7)
Mauritius	2.3 (-1.3 to 6.6)	3.2 (1.2 to 5.3)	7.7 (-2.8 to 22.7)	7.5 (-0.3 to 18.0)	7.7 (2.6 to 14.3)
Mexico	2.3 (-1.2 to 6.3)	3.1 (1.2 to 5.2)	7.5 (-2.5 to 22.0)	7.2 (-0.3 to 17.5)	7.5 (2.4 to 13.6)
Micronesia (Federated States of)	2.8 (-1.6 to 8.0)	3.9 (1.6 to 6.5)	9.3 (-3.3 to 27.5)	9.0 (-0.4 to 21.6)	9.3 (3.0 to 17.0)
Mongolia	1.5 (-0.9 to 4.2)	2.0 (0.8 to 3.4)	5.0 (-1.7 to 14.6)	4.8 (-0.2 to 11.4)	5.0 (1.6 to 9.3)
Morocco	2.1 (-1.1 to 5.8)	2.8 (1.1 to 4.9)	6.8 (-2.5 to 20.8)	6.6 (-0.3 to 15.9)	6.8 (2.2 to 12.7)
Mozambique	0.5 (-0.3 to 1.3)	0.6 (0.2 to 1.1)	1.5 (-0.5 to 4.7)	1.5 (-0.1 to 3.6)	1.5 (0.5 to 2.9)
Myanmar	0.9 (-0.5 to 2.4)	1.2 (0.5 to 2.0)	2.9 (-1.0 to 8.7)	2.8 (-0.1 to 6.9)	2.9 (0.9 to 5.4)
Namibia	2.6 (-1.5 to 7.3)	3.5 (1.4 to 6.0)	8.6 (-3.0 to 25.1)	8.3 (-0.5 to 19.4)	8.6 (2.7 to 15.6)
Nauru	3.3 (-1.8 to 9.3)	4.4 (1.8 to 7.6)	10.5 (-3.5 to 31.2)	10.2 (-0.6 to 24.5)	10.5 (3.4 to 19.4)
Nepal	1.1 (-0.6 to 3.0)	1.5 (0.6 to 2.4)	3.6 (-1.3 to 10.7)	3.5 (-0.1 to 8.3)	3.6 (1.2 to 6.6)
Netherlands	2.1 (-1.2 to 6.0)	2.9 (1.2 to 4.9)	7.1 (-2.5 to 20.9)	6.8 (-0.4 to 16.2)	7.1 (2.3 to 13.1)
New Zealand	3.3 (-2.0 to 9.3)	4.5 (1.9 to 7.4)	10.6 (-3.5 to 31.6)	10.3 (-0.5 to 24.7)	10.6 (3.5 to 19.2)
Niger	1.8 (-1.0 to 5.1)	2.4 (0.9 to 4.1)	5.9 (-2.1 to 17.9)	5.7 (-0.2 to 13.9)	5.9 (2.0 to 11.0)
Nigeria	2.1 (-1.2 to 5.9)	2.9 (1.2 to 4.9)	7.1 (-2.5 to 21.2)	6.8 (-0.2 to 16.2)	7.1 (2.4 to 12.8)
Niue	0.6 (-0.3 to 1.6)	0.8 (0.3 to 1.3)	1.9 (-0.7 to 5.7)	1.8 (-0.1 to 4.4)	1.9 (0.6 to 3.6)
Norway	2.5 (-1.3 to 7.0)	3.4 (1.4 to 5.6)	8.2 (-2.8 to 24.4)	7.9 (-0.3 to 18.6)	8.2 (2.8 to 14.9)
Oman	2.6 (-1.4 to 7.2)	3.5 (1.4 to 5.9)	8.4 (-3.0 to 25.4)	8.2 (-0.2 to 19.5)	8.4 (2.8 to 15.6)
Pakistan	2.6 (-1.5 to 7.6)	3.6 (1.3 to 6.2)	8.6 (-2.9 to 26.6)	8.3 (-0.5 to 20.0)	8.6 (2.8 to 16.6)
Palau	3.2 (-1.8 to 9.1)	4.3 (1.7 to 7.3)	10.3 (-3.8 to 31.3)	9.9 (-0.4 to 24.0)	10.3 (3.3 to 18.7)
Papua New Guinea	1.2 (-0.7 to 3.4)	1.6 (0.6 to 2.9)	4.0 (-1.4 to 11.9)	3.8 (-0.1 to 9.4)	4.0 (1.2 to 7.6)
Paraguay	2.9 (-1.7 to 8.1)	4.0 (1.6 to 6.7)	9.5 (-3.0 to 28.4)	9.2 (-0.5 to 22.0)	9.5 (3.1 to 17.2)
Philippines	3.1 (-1.8 to 8.7)	4.2 (1.6 to 7.0)	10.0 (-3.6 to 29.4)	9.7 (-0.4 to 23.0)	10.0 (3.4 to 18.2)
Poland	2.5 (-1.5 to 7.1)	3.5 (1.4 to 5.8)	8.3 (-2.9 to 24.6)	8.1 (-0.3 to 19.3)	8.3 (2.8 to 15.3)
Portugal	3.4 (-1.9 to 9.5)	4.6 (1.9 to 7.6)	10.8 (-4.0 to 31.8)	10.5 (-0.3 to 25.2)	10.8 (3.7 to 19.8)
Qatar	2.9 (-1.6 to 8.2)	3.9 (1.5 to 6.6)	9.3 (-3.4 to 27.5)	9.0 (-0.5 to 21.7)	9.3 (3.0 to 17.1)
Republic of Korea	2.8 (-1.6 to 8.1)	3.8 (1.3 to 6.8)	9.0 (-3.1 to 28.8)	8.7 (-0.2 to 21.7)	9.0 (2.6 to 17.7)
Republic of Moldova	0.9 (-0.5 to 2.6)	1.3 (0.5 to 2.2)	3.1 (-1.1 to 9.6)	3.0 (-0.1 to 7.2)	3.1 (1.0 to 5.8)
Romania	2.8 (-1.5 to 7.8)	3.8 (1.5 to 6.3)	9.0 (-3.2 to 27.5)	8.7 (-0.5 to 20.9)	9.0 (3.0 to 16.7)
Russian Federation	1.4 (-0.8 to 3.8)	1.9 (0.7 to 3.2)	4.6 (-1.6 to 13.6)	4.41 (-0.1 to 10.7)	4.6 (1.5 to 8.3)
Rwanda	1.2 (-0.7 to 3.2)	1.6 (0.6 to 2.7)	3.9 (-1.4 to 11.8)	3.8 (-0.1 to 9.1)	3.9 (1.3 to 7.3)

Saint Kitts & Nevis	2.5 (-1.4 to 7.1)	3.4 (1.3 to 5.8)	8.3 (-2.9 to 25.0)	8.0 (-0.3 to 19.0)	8.3 (2.7 to 15.4)
Saint Lucia	3.1 (-1.7 to 8.7)	4.2 (1.7 to 7.1)	10.0 (-3.4 to 30.0)	9.7 (-0.5 to 23.3)	10.0 (3.3 to 18.3)
Samoa	1.0 (-0.6 to 2.9)	1.4 (0.5 to 2.4)	3.4 (-1.2 to 10.2)	3.3 (-0.2 to 8.0)	3.4 (1.1 to 6.4)
Sao Tome & Principe	1.2 (-0.7 to 3.6)	1.7 (0.6 to 2.9)	4.2 (-1.5 to 12.4)	4.0 (-0.1 to 9.7)	4.2 (1.4 to 7.6)
Saudi Arabia	4.1 (-2.3 to 11.4)	5.5 (2.2 to 9.1)	12.9 (-4.8 to 38.2)	12.5 (-0.7 to 30.2)	12.9 (4.4 to 23.1)
Senegal	1.8 (-1.1 to 5.1)	2.5 (1.0 to 4.1)	6.1 (-2.1 to 17.7)	5.9 (-0.3 to 13.9)	6.1 (1.9 to 11.0)
Serbia	3.1 (-1.7 to 8.7)	4.2 (1.7 to 7.0)	10.0 (-3.4 to 29.7)	9.6 (-0.3 to 23.0)	10.0 (3.2 to 18.3)
Seychelles	1.5 (-0.8 to 4.2)	2.0 (0.8 to 3.5)	5.0 (-1.8 to 15.1)	4.8 (-0.2 to 11.8)	5.0 (1.6 to 9.2)
Sierra Leone	1.1 (-0.7 to 3.2)	1.6 (0.6 to 2.6)	3.9 (-1.4 to 11.3)	3.7 (-0.1 to 8.8)	3.9 (1.3 to 7.2)
Singapore	2.8 (-1.6 to 8.3)	3.9 (1.4 to 7.0)	9.3 (-3.3 to 28.8)	9.0 (-0.5 to 22.2)	9.3 (2.8 to 18.1)
Slovakia	2.7 (-1.6 to 7.6)	3.7 (1.5 to 6.2)	8.9 (-2.9 to 26.2)	8.6 (-0.4 to 20.5)	8.9 (2.9 to 16.3)
Slovenia	2.5 (-1.5 to 7.2)	3.4 (1.3 to 5.7)	8.3 (-2.9 to 24.2)	8.0 (-0.3 to 19.4)	8.3 (2.7 to 15.1)
Solomon Islands	1.4 (-0.8 to 4.2)	2.0 (0.8 to 3.4)	4.9 (-1.8 to 14.5)	4.7 (-0.2 to 11.4)	4.9 (1.6 to 9.1)
South Africa	3.0 (-1.6 to 8.4)	4.0 (1.6 to 6.7)	9.7 (-3.2 to 28.9)	9.4 (-0.5 to 22.4)	9.7 (3.2 to 17.7)
Spain	2.1 (-1.2 to 5.9)	2.9 (1.2 to 4.8)	7.0 (-2.3 to 20.6)	6.8 (-0.3 to 16.2)	7.0 (2.3 to 12.8)
Sri Lanka	2.3 (-1.2 to 6.5)	3.1 (1.2 to 5.3)	7.5 (-2.5 to 22.5)	7.2 (-0.4 to 17.3)	7.5 (2.4 to 13.7)
State of Palestine	3.1 (-1.7 to 8.9)	4.3 (1.7 to 7.1)	10.2 (-3.5 to 30.3)	9.9 (-0.5 to 23.7)	10.2 (3.3 to 18.7)
Suriname	3.4 (-2.0 to 9.9)	4.7 (1.9 to 7.8)	11.1 (-3.9 to 32.8)	10.7 (-0.3 to 25.5)	11.1 (3.6 to 20.0)
Swaziland	2.2 (-1.3 to 6.3)	3.0 (1.2 to 5.0)	7.3 (-2.6 to 21.1)	7.0 (-0.3 to 16.7)	7.3 (2.5 to 13.3)
Sweden	1.8 (-1.1 to 5.1)	2.5 (1.0 to 4.2)	6.1 (-2.1 to 18.6)	5.9 (-0.4 to 14.1)	6.1 (1.9 to 11.0)
Switzerland	1.9 (-1.1 to 5.4)	2.5 (1.0 to 4.3)	6.2 (-2.0 to 18.2)	6.0 (-0.3 to 14.2)	6.2 (2.0 to 11.4)
Tajikistan	2.3 (-1.3 to 6.4)	3.1 (1.2 to 5.4)	7.6 (-2.6 to 22.9)	7.3 (-0.3 to 17.9)	7.6 (2.4 to 14.2)
Thailand	1.9 (-1.1 to 5.6)	2.6 (1.0 to 4.5)	6.4 (-2.2 to 18.9)	6.2 (-0.2 to 15.1)	6.4 (2.1 to 12.0)
Timor-Leste	1.4 (-0.8 to 4.1)	1.9 (0.7 to 3.4)	4.8 (-1.7 to 14.4)	4.6 (-0.3 to 11.2)	4.8 (1.5 to 9.0)
Togo	0.8 (-0.4 to 2.2)	1.1 (0.4 to 1.8)	2.7 (-0.9 to 7.8)	2.6 (-0.1 to 6.2)	2.7 (0.9 to 4.9)
Tokelau	0.9 (-0.5 to 2.6)	1.2 (0.5 to 2.1)	3.0 (-1.0 to 9.1)	2.9 (-0.1 to 7.2)	3.0 (1.0 to 5.7)
Tonga	1.4 (-0.7 to 4.0)	1.9 (0.7 to 3.2)	4.7 (-1.6 to 14.1)	4.5 (-0.2 to 10.7)	4.7 (1.5 to 8.8)
Trinidad and Tobago	3.0 (-1.7 to 8.4)	4.0 (1.6 to 7.0)	9.7 (-3.5 to 28.5)	9.4 (-0.1 to 22.3)	9.7 (3.0 to 17.8)
Tunisia	2.4 (-1.4 to 6.8)	3.2 (1.2 to 5.4)	7.8 (-2.8 to 23.2)	7.6 (-0.4 to 18.1)	7.8 (2.5 to 14.3)
Turkey	2.4 (-1.4 to 6.8)	3.3 (1.3 to 5.6)	7.9 (-2.8 to 23.2)	7.6 (-0.5 to 17.9)	7.9 (2.5 to 14.6)
Tuvalu	2.1 (-1.2 to 6.0)	2.9 (1.2 to 5.0)	7.1 (-2.4 to 21.2)	6.9 (-0.3 to 16.5)	7.1 (2.3 to 13.1)
Uganda	0.4 (-0.3 to 1.3)	0.6 (0.2 to 1.0)	1.5 (-0.5 to 4.4)	1.5 (-0.1 to 3.5)	1.5 (0.5 to 2.8)
Ukraine	1.5 (-0.9 to 4.4)	2.1 (0.8 to 3.6)	5.2 (-1.7 to 15.4)	5.0 (-0.2 to 11.9)	5.2 (1.7 to 9.7)
United Arab Emirates	3.2 (-1.8 to 9.1)	4.4 (1.7 to 7.3)	10.4 (-3.5 to 30.7)	10.1 (-0.4 to 23.9)	10.4 (3.4 to 19.2)
United Kingdom	2.8 (-1.6 to 7.9)	3.8 (1.5 to 6.3)	9.1 (-3.4 to 27.0)	8.8 (-0.4 to 20.9)	9.1 (3.0 to 16.4)
United Republic of Tanzania	0.5 (-0.3 to 1.4)	0.7 (0.3 to 1.2)	1.8 (-0.6 to 5.4)	1.7 (-0.1 to 4.1)	1.8 (0.6 to 3.3)
United States of America	3.1 (-1.9 to 8.8)	4.2 (1.7 to 7.1)	10.1 (-3.6 to 30.1)	9.8 (-0.5 to 23.3)	10.1 (3.4 to 18.3)
Uruguay	1.8 (-1.1 to 5.0)	2.4 (1.0 to 4.1)	5.9 (-2.2 to 17.9)	5.7 (-0.2 to 13.6)	5.9 (1.9 to 10.8)
Uzbekistan	1.5 (-0.8 to 4.3)	2.1 (0.8 to 3.6)	5.1 (-1.8 to 15.3)	4.9 (-0.2 to 11.8)	5.1 (1.6 to 9.5)
Vanuatu	0.6 (-0.4 to 1.9)	0.9 (0.3 to 1.5)	2.2 (-0.7 to 6.6)	2.1 (-0.1 to 5.2)	2.2 (0.7 to 4.2)
Venezuela (Bolivarian Republic of)	2.5 (-1.3 to 7.1)	3.3 (1.4 to 5.7)	8.1 (-2.8 to 23.7)	7.8 (-0.3 to 18.9)	8.1 (2.7 to 14.2)
Viet Nam	2.0 (-1.1 to 5.7)	2.7 (1.1 to 4.6)	6.6 (-2.4 to 19.7)	6.4 (0.0 to 15.2)	6.6 (2.2 to 12.3)
Zambia	1.7 (-1.0 to 4.9)	2.4 (0.9 to 4.0)	5.8 (-2.2 to 17.2)	5.6 (-0.3 to 13.5)	5.8 (1.9 to 10.3)

Zimbabwe	2.1 (-1.2 to 5.9)	2.9 (1.1 to 4.8)	7.0 (-2.6 to 20.7)	6.8 (-0.4 to 16.2)	7.0 (2.3 to 12.81)

Supplementary Table 3. Population Attributable Risks for Physical Inactivity and Female Cancer Outcomes by Country

Country	Breast Cancer	Endometrial Cancer
Algeria	3.5 (1.5 to 5.7)	3.5 (-0.6 to 8.1)
American Samoa	4.9 (2.2 to 8.0)	4.9 (-0.9 to 11.3)
Andorra	3.5 (1.6 to 5.6)	3.5 (-0.6 to 8.0)
Argentina	3.9 (1.8 to 6.4)	3.9 (-0.7 to 9.0)
Armenia	2.0 (0.8 to 3.3)	2.0 (-0.3 to 4.5)
Australia	2.9 (1.3 to 4.8)	2.9 (-0.5 to 6.7)
Austria	2.9 (1.3 to 4.7)	2.9 (-0.5 to 6.7)
Bahamas	4.8 (2.1 to 7.6)	4.8 (-0.7 to 11.0)
Bangladesh	3.4 (1.4 to 5.9)	3.4 (-0.6 to 8.3)
Barbados	4.7 (2.1 to 7.6)	4.7 (-0.9 to 10.9)
Belarus	1.3 (0.5 to 2.2)	1.3 (-0.2 to 3.1)
Belgium	3.5 (1.6 to 5.7)	3.5 (-0.5 to 8.2)
Benin	1.6 (0.7 to 2.6)	1.6 (-0.3 to 3.7)
Bermuda	2.8 (1.2 to 4.6)	2.8 (-0.4 to 6.6)
Bhutan	2.6 (1.1 to 4.2)	2.6 (-0.5 to 6.1)
Bosnia & Herzegovina	2.5 (1.0 to 4.1)	2.5 (-0.3 to 5.8)
Botswana	2.3 (1.0 to 3.8)	2.3 (-0.4 to 5.4)
Brazil	4.6 (2.0 to 7.4)	4.6 (-0.7 to 10.6)
British Virgin Islands	3.1 (1.4 to 5.1)	3.1 (-0.6 to 7.3)
Brunei Darussalam	3.0 (1.2 to 5.0)	3.0 (-0.4 to 6.9)
Bulgaria	3.6 (1.6 to 5.9)	3.6 (-0.6 to 8.3)
Burkina Faso	2.0 (0.9 to 3.3)	2.0 (-0.3 to 4.7)
Cabo Verde	2.2 (1.0 to 3.6)	2.2 (-0.3 to 5.1)
Cambodia	1.0 (0.4 to 1.7)	1.0 (-0.1 to 2.4)
Cameroon	3.1 (1.4 to 5.0)	3.1 (-0.6 to 7.2)
Canada	2.8 (1.2 to 4.5)	2.8 (-0.4 to 6.5)
Cayman Islands	3.3 (1.5 to 5.4)	3.3 (-0.5 to 7.7)
Central African Republic	1.4 (0.6 to 2.3)	1.4 (-0.2 to 3.3)
Chad	2.4 (1.0 to 3.9)	2.4 (-0.4 to 5.5)
Chile	2.5 (1.1 to 4.1)	2.5 (-0.4 to 5.9)
China	1.1 (0.5 to 1.8)	1.1 (-0.2 to 2.6)
Colombia	4.2 (1.9 to 6.8)	4.2 (-0.6 to 9.6)
Comoros	1.7 (0.7 to 2.8)	1.7 (-0.3 to 4.0)
Congo	2.7 (1.2 to 4.5)	2.7 (-0.4 to 6.5)
Cook Islands	2.4 (1.0 to 4.0)	2.4 (-0.4 to 5.7)
Costa Rica	4.7 (2.1 to 7.5)	4.7 (-0.7 to 10.8)
Cote d'Ivoire	3.3 (1.5 to 5.2)	3.3 (-0.5 to 7.5)
Croatia	3.1 (1.4 to 5.1)	3.1 (-0.5 to 7.3)
Cuba	3.7 (1.6 to 6.0)	3.7 (-0.6 to 8.5)
Cyprus	4.4 (1.9 to 7.0)	4.4 (-0.7 to 10.1)

Czech Republic	3.0 (1.3 to 4.8)	3.0 (-0.5 to 7.0)
Democratic Republic of the Congo	2.4 (1.0 to 3.8)	2.4 (-0.4 to 5.4)
Denmark	2.7 (1.2 to 4.5)	2.7 (-0.4 to 6.4)
Dominica	2.6 (1.2 to 4.3)	2.6 (-0.4 to 6.1)
Dominican Republic	3.8 (1.6 to 6.1)	3.8 (-0.6 to 8.7)
Ecuador	2.7 (1.1 to 4.3)	2.6 (-0.4 to 6.1)
Egypt	3.4 (1.5 to 5.5)	3.4 (-0.6 to 7.9)
Eritrea	2.7 (1.2 to 4.3)	2.7 (-0.4 to 6.2)
Estonia	3.0 (1.4 to 5.0)	3.0 (-0.5 to 7.0)
Ethiopia	1.6 (0.7 to 2.6)	1.6 (-0.3 to 3.8)
Fiji	2.1 (0.9 to 3.5)	2.2 (-0.4 to 5.0)
Finland	1.4 (0.6 to 2.3)	1.4 (-0.2 to 3.3)
France	3.0 (1.3 to 4.8)	3.0 (-0.5 to 6.9)
French Polynesia	2.0 (0.8 to 3.3)	2.0 (-0.3 to 4.7)
Gabon	2.9 (1.3 to 4.8)	2.9 (-0.5 to 6.8)
Gambia	2.3 (1.0 to 3.8)	2.3 (-0.3 to 5.3)
Georgia	1.7 (0.7 to 2.8)	1.7 (-0.3 to 3.9)
Germany	3.8 (1.7 to 6.1)	3.8 (-0.6 to 8.7)
Ghana	2.2 (1.0 to 3.5)	2.2 (-0.4 to 5.1)
Greece	3.6 (1.6 to 5.8)	3.6 (-0.5 to 8.3)
Grenada	3.1 (1.4 to 5.1)	3.1 (-0.6 to 7.2)
Guatemala	3.2 (1.4 to 5.2)	3.2 (-0.5 to 7.6)
Guinea	1.6 (0.7 to 2.7)	1.6 (-0.3 to 3.9)
Hungary	3.8 (1.7 to 6.1)	3.8 (-0.6 to 8.7)
India	3.8 (1.6 to 6.5)	3.8 (-0.5 to 9.2)
Indonesia	1.9 (0.8 to 3.3)	1.9 (-0.3 to 4.6)
Iran (Islamic Republic of)	3.8 (1.7 to 6.2)	3.8 (-0.5 to 8.8)
Iraq	5.5 (2.5 to 8.8)	5.5 (-0.8 to 12.7)
Ireland	3.2 (1.4 to 5.2)	3.2 (-0.4 to 7.5)
Italy	4.0 (1.8 to 6.4)	4.0 (-0.5 to 9.3)
Jamaica	3.2 (1.4 to 5.3)	3.2 (-0.5 to 7.5)
Japan	3.2 (1.3 to 5.8)	3.2 (-0.5 to 8.0)
Jordan	1.2 (0.5 to 2.0)	1.2 (-0.2 to 2.8)
Kazakhstan	2.5 (1.1 to 4.2)	2.5 (-0.4 to 5.9)
Kenya	1.5 (0.7 to 2.4)	1.5 (-0.2 to 3.5)
Kiribati	4.0 (1.7 to 6.4)	4.0 (-0.6 to 9.2)
Kuwait	6.3 (2.8 to 10.1)	6.3 (-1.1 to 14.5)
Kyrgyzstan	1.5 (0.6 to 2.6)	1.5 (-0.2 to 3.5)
Lao People's Democratic Republic	1.8 (0.8 to 3.0)	1.8 (-0.3 to 4.4)
Latvia	2.9 (1.3 to 4.7)	2.9 (-0.4 to 6.7)
Lebanon	2.9 (1.2 to 4.7)	2.9 (-0.5 to 6.7)
Lesotho	0.5 (0.2 to 0.9)	0.5 (-0.1 to 1.3)
Liberia	2.5 (1.1 to 4.1)	2.5 (-0.4 to 5.8)

Libya	3.6 (1.6 to 5.9)	3.6 (-0.5 to 8.3)
Lithuania	2.6 (1.1 to 4.2)	2.6 (-0.4 to 6.0)
Luxembourg	2.7 (1.2 to 4.3)	2.7 (-0.4 to 6.2)
Madagascar	1.9 (0.8 to 3.1)	1.9 (-0.3 to 4.4)
Malawi	1.6 (0.7 to 2.6)	1.6 (-0.2 to 3.7)
Malaysia	3.7 (1.7 to 6.1)	3.7 (-0.7 to 8.7)
Maldives	3.1 (1.3 to 5.1)	3.0 (-0.5 to 7.2)
Mali	4.1 (1.9 to 6.4)	4.1 (-0.8 to 9.3)
Malta	4.1 (1.8 to 6.5)	4.1 (-0.6 to 9.5)
Marshall Islands	4.3 (1.9 to 7.0)	4.3 (-0.7 to 10.1)
Mauritania	4.0 (1.8 to 6.4)	4.0 (-0.7 to 9.2)
Mauritius	2.8 (1.2 to 4.6)	2.8 (-0.4 to 6.5)
Mexico	2.8 (1.3 to 4.6)	2.8 (-0.5 to 6.5)
Micronesia (Federated States of)	3.5 (1.5 to 5.8)	3.5 (-0.6 to 8.2)
Mongolia	1.7 (0.8 to 2.8)	1.7 (-0.2 to 4.1)
Morocco	2.8 (1.2 to 4.6)	2.8 (-0.5 to 6.5)
Mozambique	0.5 (0.2 to 0.9)	0.5 (-0.1 to 1.3)
Myanmar	1.2 (0.5 to 2.0)	1.2 (-0.2 to 2.8)
Namibia	3.3 (1.4 to 5.3)	3.3 (-0.5 to 7.6)
Nauru	4.3 (1.9 to 6.9)	4.3 (-0.7 to 10.0)
Nepal	1.3 (0.6 to 2.1)	1.3 (-0.2 to 3.0)
Netherlands	2.5 (1.1 to 4.1)	2.5 (-0.4 to 5.9)
New Zealand	3.9 (1.7 to 6.3)	3.9 (-0.7 to 9.0)
Niger	2.2 (1.0 to 3.7)	2.2 (-0.4 to 5.2)
Nigeria	2.6 (1.1 to 4.2)	2.6 (-0.5 to 6.1)
Niue	0.5 (0.2 to 0.9)	0.5 (-0.1 to 1.3)
Norway	3.0 (1.3 to 4.8)	3.0 (-0.4 to 6.8)
Oman	3.5 (1.5 to 5.7)	3.5 (-0.6 to 8.1)
Pakistan	3.8 (1.5 to 6.4)	3.8 (-0.5 to 9.0)
Palau	4.6 (2.0 to 7.5)	4.6 (-0.6 to 10.7)
Papua New Guinea	1.6 (0.6 to 2.8)	1.6 (-0.2 to 3.9)
Paraguay	3.2 (1.4 to 5.2)	3.2 (-0.4 to 7.4)
Philippines	4.2 (1.9 to 6.8)	4.2 (-0.7 to 9.8)
Poland	2.9 (1.3 to 4.8)	2.9 (-0.5 to 6.8)
Portugal	4.2 (1.9 to 6.8)	4.2 (-0.7 to 9.6)
Qatar	4.2 (1.9 to 6.8)	4.2 (-0.7 to 9.8)
Republic of Korea	3.6 (1.4 to 6.2)	3.6 (-0.6 to 8.7)
Republic of Moldova	1.0 (0.4 to 1.6)	1.0 (-0.2 to 2.3)
Romania	3.3 (1.5 to 5.4)	3.3 (-0.6 to 7.7)
Russian Federation	1.6 (0.7 to 2.6)	1.6 (-0.3 to 3.6)
Rwanda	1.6 (0.7 to 2.6)	1.6 (-0.3 to 3.6)
Saint Kitts & Nevis	3.5 (1.5 to 5.7)	3.5 (-0.6 to 8.2)
Saint Lucia	4.5 (2.0 to 7.3)	4.5 (-0.8 to 10.4)

Samoa	1.5 (0.6 to 2.6)	1.5 (-0.2 to 3.7)
Sao Tome & Principe	1.9 (0.8 to 3.0)	1.9 (-0.3 to 4.3)
Saudi Arabia	5.5 (2.5 to 8.8)	5.5 (-0.9 to 12.7)
Senegal	2.5 (1.1 to 4.0)	2.5 (-0.3 to 5.7)
Serbia	3.8 (1.7 to 6.2)	3.8 (-0.5 to 8.7)
Seychelles	1.8 (0.8 to 3.0)	1.8 (-0.3 to 4.3)
Sierra Leone	1.6 (0.7 to 2.7)	1.6 (-0.3 to 3.8)
Singapore	3.4 (1.3 to 5.8)	3.4 (-0.5 to 8.3)
Slovakia	3.3 (1.5 to 5.4)	3.3 (-0.5 to 7.7)
Slovenia	3.2 (1.4 to 5.2)	3.2 (-0.5 to 7.4)
Solomon Islands	2.1 (0.9 to 3.4)	2.1 (-0.3 to 4.8)
South Africa	4.1 (1.8 to 6.6)	4.1 (-0.6 to 9.4)
Spain	2.7 (1.2 to 4.4)	2.7 (-0.4 to 6.2)
Sri Lanka	3.2 (1.4 to 5.3)	3.2 (-0.5 to 7.5)
State of Palestine	4.2 (1.9 to 6.8)	4.2 (-0.6 to 9.7)
Suriname	4.4 (1.9 to 7.0)	4.4 (-0.7 to 10.1)
Swaziland	2.8 (1.2 to 4.6)	2.8 (-0.5 to 6.5)
Sweden	2.2 (0.9 to 3.6)	2.2 (-0.3 to 5.0)
Switzerland	2.3 (1.0 to 3.7)	2.3 (-0.3 to 5.3)
Tajikistan	3.4 (1.5 to 5.6)	3.4 (-0.7 to 7.9)
Thailand	2.4 (1.0 to 4.0)	2.4 (-0.4 to 5.7)
Timor-Leste	2.2 (0.9 to 3.8)	2.2 (-0.3 to 5.3)
Togo	0.9 (0.4 to 1.5)	0.9 (-0.1 to 2.2)
Tokelau	1.6 (0.6 to 2.7)	1.6 (-0.2 to 3.7)
Tonga	2.3 (1.0 to 3.8)	2.3 (-0.4 to 5.4)
Trinidad and Tobago	4.2 (1.9 to 6.9)	4.2 (-0.8 to 9.9)
Tunisia	3.0 (1.3 to 4.9)	3.0 (-0.5 to 7.0)
Turkey	3.4 (1.5 to 5.5)	3.4 (-0.6 to 7.9)
Tuvalu	3.2 (1.4 to 5.3)	3.2 (-0.6 to 7.5)
Uganda	0.5 (0.2 to 0.9)	0.5 (-0.1 to 1.2)
Ukraine	1.8 (0.8 to 3.0)	1.8 (-0.3 to 4.2)
United Arab Emirates	4.3 (1.9 to 6.8)	4.3 (-0.6 to 9.9)
United Kingdom	3.5 (1.6 to 5.6)	3.5 (-0.6 to 8.0)
United Republic of Tanzania	0.6 (0.3 to 1.1)	0.6 (-0.1 to 1.5)
United States of America	4.1 (1.9 to 6.6)	4.1 (-0.6 to 9.6)
Uruguay	2.3 (1.0 to 3.8)	2.3 (-0.4 to 5.3)
Uzbekistan	2.2 (0.9 to 3.7)	2.2 (-0.3 to 5.2)
Vanuatu	0.8 (0.3 to 1.3)	0.8 (-0.1 to 1.9)
Venezuela (Bolivarian Republic of)	2.9 (1.3 to 4.8)	2.9 (-0.4 to 6.8)
Viet Nam	2.7 (1.2 to 4.4)	2.7 (-0.4 to 6.4)
Zambia	2.2 (1.0 to 3.6)	2.2 (-0.4 to 5.1)
Zimbabwe	2.7 (1.2 to 4.4)	2.7 (-0.4 to 6.3)

Supplementary Table 4. Population Attributable Risks for Physical Inactivity and Type 2 Diabetes, Dementia and Depression by Country

Country	Type 2 Diabetes	Dementia	Depression
Algeria	5.4 (3.6 to 7.5)	9.7 (2.9 to 18.5)	8.6 (1.5 to 17.7)
American Samoa	8.3 (5.7 to 11.4)	14.6 (4.5 to 27.4)	13.0 (2.2 to 26.7)
Andorra	6.1 (4.1 to 8.3)	10.9 (3.4 to 20.7)	9.7 (1.6 to 20.0)
Argentina	6.6 (4.4 to 9.0)	11.8 (3.6 to 22.1)	10.4 (1.8 to 21.3)
Armenia	3.7 (2.3 to 5.3)	6.7 (2.0 to 2.8)	6.0 (1.2 to 12.3)
Australia	4.9 (3.3 to 6.8)	8.9 (2.6 to16.7)	7.8 (1.4 to 16.1)
Austria	4.9 (3.3 to 6.6)	8.8 (2.7 to 16.5)	7.8 (1.4 to 16.0)
Bahamas	6.9 (4.6 to 9.4)	12.2 (3.7 to 23.0)	10.8 (1.9 to 22.0)
Bangladesh	4.5 (2.6 to 6.7)	8.2 (2.3 to 15.9)	7.2 (1.2 to 15.3)
Barbados	6.8 (4.6 to 9.3)	12.1 (3.7 to 22.8)	10.7 (1.9 to 21.7)
Belarus	2.3 (1.5 to 3.4)	4.3 (1.2 to 8.3)	3.8 (0.6 to 7.9)
Belgium	5.7 (4.0 to 7.7)	10.3 (2.9 to 19.1)	9.1 (1.7 to 18.3)
Benin	2.6 (1.7 to 3.7)	4.8 (1.4 to 9.1)	4.3 (0.8 to 8.7)
Bermuda	4.3 (2.8 to 6.0)	7.7 (2.4 to 14.9)	6.8 (1.1 to 14.2)
Bhutan	3.8 (2.6 to 5.1)	6.9 (2.1 to 12.8)	6.1 (1.1 to 12.4)
Bosnia & Herzegovina	4.2 (2.6 to 5.9)	7.5 (2.2 to 14.4)	6.7 (1.2 to 13.9)
Botswana	3.6 (2.4 to 4.9)	6.5 (2.0 to 12.1)	5.7 (1.0 to 11.8)
Brazil	7.4 (5.1 to 9.9)	13.1 (3.9 to 24.7)	11.6 (2.1 to 23.3)
British Virgin Islands	4.5 (2.9 to 6.2)	8.1 (2.4 to 15.2)	7.1 (1.3 to 14.7)
Brunei Darussalam	4.4 (2.7 to 6.5)	8.0 (2.4 to 15.5)	7.1 (1.2 to 15.0)
Bulgaria	6.2 (4.1 to 8.4)	11.0 (3.2 to 20.4)	9.8 (1.8 to 20.1)
Burkina Faso	3.3 (2.2 to 4.7)	6.1 (1.8 to 11.5)	5.4 (0.9 to 11.2)
Cabo Verde	3.2 (2.1 to 4.5)	5.9 (1.8 to 11.3)	5.2 (0.8 to 10.8)
Cambodia	1.8 (1.0 to 2.6)	3.3 (0.9 to 6.4)	2.9 (0.5 to 6.1)
Cameroon	4.6 (3.1 to 6.3)	8.4 (2.6 to 15.5)	7.4 (1.3 to 15.1)
Canada	4.6 (3.1 to 6.4)	8.4 (2.5 to 15.8)	7.4 (1.3 to 15.3)
Cayman Islands	4.7(3.1 to 6.6)	8.5 (2.6 to 16.3)	7.5 (1.2 to 15.5)
Central African Republic	2.4 (1.6 to 3.3)	4.4 (1.3 to 8.2)	3.9 (0.7 to 7.9)
Chad	3.8 (2.5 to 5.4)	6.9 (2.1 to 13.2)	6.1 (1.1 to 12.5)
Chile	4.3 (2.8 to 6.1)	7.8 (2.3 to 14.8)	6.9 (1.2 to 14.4)
China	2.3 (1.5 to 3.4)	4.3 (1.2 to 8.4)	3.8 (0.6 to 8.0)
Colombia	7.0 (4.7 to 9.5)	12.3 (3.8 to 23.0)	11.0 (2.2 to 22.6)
Comoros	2.4 (1.6 to 3.3)	4.4 (1.3 to 8.4)	3.9 (0.7 to 7.9)
Congo	4.5 (3.0 to 6.3)	8.2 (2.7 to 15.7)	7.3 (1.2 to 14.8)
Cook Islands	3.1 (1.9 to 4.4)	5.6 (1.7 to 10.6)	4.9 (0.9 to 10.3)
Costa Rica	7.3 (5.0 to 9.8)	12.9 (3.8 to 23.8)	11.4 (1.9 to 23.7)
Cote d'Ivoire	5.3 (3.6 to 7.2)	9.6 (2.8 to 18.1)	8.5 (1.6 to 17.4)
Croatia	5.0 (3.3 to 7.0)	9.1 (2.7 to 17.1)	8.0 (1.4 to 16.6)
Cuba	5.9 (4.0 to 8.1)	10.6 (3.2 to 19.6)	9.4 (1.6 to 19.4)

Cyprus	7.0 (4.8 to 9.5)	12.4 (3.8 to 23.1)	11.1 (2.0 to 22.5)
Czech Republic	5.0 (3.4 to 7.0)	9.1 (2.6 to 17.1)	8.0 (1.5 to 16.4)
Democratic Republic of the Congo	3.9 (2.6 to 5.3)	7.1 (2.1 to 13.2)	6.3 (1.0 to 12.7)
Denmark	4.6 (3.1 to 6.4)	8.4 (2.6 to 15.5)	7.4 (1.3 to 15.1)
Dominica	3.5 (2.3 to 5.0)	6.5 (1.9 to 12.4)	5.7 (1.0 to 11.8)
Dominican Republic	6.2 (4.2 to 8.5)	11.1 (3.5 to 20.9)	9.8 (1.7 to 19.9)
Ecuador	4.4 (2.9 to 6.2)	8.0 (2.5 to 15.1)	7.1 (1.2 to 14.6)
Egypt	5.0 (3.3 to 7.0)	9.0 (2.7 to 17.0)	8.0 (1.5 to 16.5)
Eritrea	3.7 (2.5 to 4.9)	6.7 (2.1 to 12.5)	5.9 (1.1 to 11.9)
Estonia	5.2 (3.4 to 7.1)	9.3 (2.8 to 17.5)	8.2 (1.3 to 16.9)
Ethiopia	2.5 (1.7 to 3.3)	4.6 (1.4 to 8.4)	4.0 (0.6 to 8.1)
Fiji	2.9 (1.8 to 4.1)	5.3 (1.6 to 10.0)	4.7 (0.8 to 9.8)
Finland	2.7 (1.8 to 3.8)	5.0 (1.5 to 9.4)	4.4 (0.8 to 9.1)
France	4.7 (3.2 to 6.5)	8.6 (2.6 to 16.0)	7.6 (1.3 to 15.7)
French Polynesia	3.0 (1.8 to 4.3)	5.4 (1.7 to 10.3)	4.8 (0.8 to 9.9)
Gabon	4.1 (2.6 to 5.9)	7.5 (2.2 to 14.3)	6.6 (1.1 to 13.8)
Gambia	3.5 (2.3 to 4.8)	6.3 (1.9 to 11.9)	5.6 (0.9 to 11.6)
Georgia	3.0 (1.9 to 4.3)	5.5 (1.7 to 10.4)	4.8 (0.7 to 10.1)
Germany	6.7 (4.6 to 9.1)	11.9 (3.6 to 22.2)	10.6 (1.8 to 21.4)
Ghana	3.6 (2.4 to 4.9)	6.5 (2.0 to 12.1)	5.8 (1.0 to 11.8)
Greece	6.0 (4.1 to 8.1)	10.8 (3.4 to 20.0)	9.6 (2.0 to 19.6)
Grenada	4.7 (3.0 to 6.5)	8.4 (2.4 to 16.0)	7.4 (1.2 to 15.4)
Guatemala	5.9 (3.9 to 8.3)	10.6 (3.2 to 20.1)	9.4 (1.6 to 19.4)
Guinea	2.4 (1.6 to 3.4)	4.4 (1.3 to 8.4)	3.9 (0.7 to 8.1)
Hungary	6.1 (4.1 to 8.3)	11.0 (3.3 to 20.4)	9.7 (1.8 to 20.3)
India	5.5 (3.3 to 8.0)	9.8 (2.7 to 19.0)	8.7 (1.5 to 18.5)
Indonesia	3.7 (2.3 to 5.3)	6.7 (2.1 to 13.0)	6.0 (1.0 to 12.5)
Iran (Islamic Republic of)	5.3 (3.6 to 7.4)	9.6 (2.8 to 18.2)	8.5 (1.5 to 17.4)
Iraq	8.1 (5.6 to 10.9)	14.3 (4.3 to 26.5)	12.7 (2.4 to 25.9)
Ireland	5.3 (3.6 to 7.2)	9.5 (2.9 to 17.6)	8.4 (1.5 to 17.2)
Italy	6.6 (4.5 to 8.9)	11.7 (3.5 to 21.9)	10.4 (1.9 to 21.1)
Jamaica	5.3 (3.5 to 7.2)	9.5 (3.0 to 17.8)	8.4 (1.3 to 17.5)
Japan	5.7 (3.2 to 8.8)	10.2 (2.8 to 20.8)	9.0 (1.4 to 19.8)
Jordan	2.0 (1.2 to 2.8)	3.7 (1.1 to 7.0)	3.2 (0.5 to 6.7)
Kazakhstan	4.5 (2.9 to 6.5)	8.1 (2.4 to 15.4)	7.2 (1.4 to 14.8)
Kenya	2.6 (1.8 to 3.4)	4.7 (1.4 to 8.6)	4.1 (0.7 to 8.3)
Kiribati	6.4 (4.2 to 8.9)	11.5 (3.4 to 21.4)	10.2 (1.8 to 21.0)
Kuwait	10.2 (7.1 to 13.5)	17.7 (5.4 to 32.8)	15.8 (3.1 to 32.4)
Kyrgyzstan	2.31 (1.4 to 3.5)	4.3 (1.2 to 8.4)	3.8 (0.6 to 8.1)
Lao People's Democratic Republic	2.7 (1.8 to 3.7)	5.0 (1.5 to 9.3)	4.4 (0.8 to 8.9)
Latvia	4.8 (3.2 to 6.7)	8.6 (2.6 to 16.1)	7.6 (1.3 to 15.7)
Lebanon	5.8 (3.9 to 8.0)	10.4 (3.2 to 19.6)	9.3 (1.7 to 19.0)
Lesotho	1.1 (0.7 to 1.5)	2.0 (0.6 to 3.8)	1.7 (0.3 to 3.7)

Liberia	4.1 (2.7 to 5.7)	7.4 (2.2 to 13.9)	6.6 (1.2 to 13.5)
Libya	5.8 (3.8 to 8.1)	10.4 (3.3 to 19.6)	9.3 (1.6 to 19.0)
Lithuania	4.3 (2.8 to 6.0)	7.8 (2.4 to 14.7)	6.9 (1.2 to 14.1)
Luxembourg	4.6 (3.1 to 6.4)	8.3 (2.6 to 15.6)	7.4 (1.3 to 14.9)
Madagascar	2.8 (1.9 to 4.0)	5.2 (1.6 to 9.8)	4.6 (0.8 to 9.6)
Malawi	2.6 (1.8 to 3.5)	4.8 (1.5 to 8.9)	4.2 (0.7 to 8.5)
Malaysia	6.2 (4.1 to 8.6)	11.0 (3.3 to 21.0)	9.8 (1.8 to 20.3)
Maldives	4.9 (3.1 to 6.9)	8.8 (2.6 to 17.1)	7.8 (1.2 to 16.2)
Mali	6.4 (4.4 to 8.6)	11.5 (3.6 to 21.1)	10.2 (1.8 to 20.8)
Malta	6.6 (4.5 to 9.0)	11.8 (3.5 to 22.0)	10.5 (1.6 to 21.1)
Marshall Islands	6.9 (4.6 to 9.5)	12.2 (3.6 to 23.2)	10.9 (2.0 to 22.6)
Mauritania	6.6 (4.5 to 8.9)	11.7 (3.7 to 21.8)	10.4 (1.9 to 20.8)
Mauritius	4.8 (3.2 to 6.7)	8.7 (2.6 to 16.6)	7.7 (1.3 to 15.9)
Mexico	4.7 (3.1 to 6.4)	8.5 (2.5 to 15.9)	7.5 (1.4 to 15.3)
Micronesia (Federated States of)	5.9 (3.9 to 8.2)	10.5 (3.1 to 20.0)	9.3 (1.7 to 19.3)
Mongolia	3.1 (2.0 to 4.3)	5.6 (1.7 to 10.7)	5.0 (0.9 to 10.2)
Morocco	4.3 (2.7 to 6.1)	7.7 (2.3 to 15.2)	6.8 (1.2 to 14.4)
Mozambique	0.9 (0.6 to 1.3)	1.8 (0.5 to 3.4)	1.5 (0.3 to 3.2)
Myanmar	1.8 (1.1 to 2.6)	3.3 (1.0 to 6.3)	2.9 (0.5 to 6.1)
Namibia	5.4 (3.6 to 7.4)	9.7 (3.0 to 17.9)	8.6 (1.4 to 17.4)
Nauru	6.7 (4.4 to 9.3)	11.9 (3.6 to 22.7)	10.5 (1.8 to 21.8)
Nepal	2.2 (1.5 to 3.0)	4.1 (1.2 to 7.6)	3.6 (0.7 to 7.3)
Netherlands	4.4 (3.0 to 6.1)	8.0 (2.3 to 15.1)	7.1 (1.2 to 14.6)
New Zealand	6.7 (4.6 to 9.0)	12.0 (3.6 to 22.3)	10.6 (1.9 to 21.8)
Niger	3.7 (2.4 to 5.2)	6.7 (2.0 to 12.9)	5.9 (0.9 to 12.4)
Nigeria	4.4 (2.9 to 6.0)	8.0 (2.4 to 15.0)	7.1 (1.3 to 14.6)
Niue	1.2 (0.7 to 1.7)	2.2 (0.6 to 4.2)	1.9 (0.3 to 3.9)
Norway	5.1 (3.4 to 7.0)	9.2 (2.7 to 17.5)	8.2 (1.4 to 16.7)
Oman	5.3 (3.5 to 7.4)	9.5 (2.9 to 18.1)	8.4 (1.4 to 17.3)
Pakistan	5.4 (3.2 to 8.0)	9.7 (2.8 to 19.1)	8.6 (1.4 to 18.5)
Palau	6.5 (4.3 to 9.0)	11.6 (3.5 to 22.0)	10.3 (1.9 to 21.1)
Papua New Guinea	2.5 (1.4 to 3.7)	4.5 (1.3 to 8.8)	4.0 (0.7 to 8.6)
Paraguay	6.0 (4.0 to 8.2)	10.7 (3.2 to 20.3)	9.5 (1.6 to 19.7)
Philippines	6.3 (4.2 to 8.6)	11.3 (3.4 to 21.2)	10.0 (1.8 to 20.4)
Poland	5.2 (3.5 to 7.2)	9.4 (2.9 to 17.9)	8.3 (1.4 to 17.1)
Portugal	6.9 (4.7 to 9.4)	12.2 (3.8 to 22.9)	10.8 (1.8 to 22.3)
Qatar	5.9 (3.9 to 8.1)	10.5 (3.1 to 19.9)	9.3 (1.6 to 19.4)
Republic of Korea	5.7 (3.1 to 8.6)	10.2 (2.8 to 20.2)	9.0 (1.4 to 19.6)
Republic of Moldova	1.9 (1.2 to 2.8)	3.6 (1.0 to 6.8)	3.1 (0.5 to 6.6)
Romania	5.7 (3.8 to 7.8)	10.2 (3.0 to 18.9)	9.0 (1.5 to 18.5)
Russian Federation	2.8 (1.8 to 4.0)	5.2 (1.5 to 9.9)	4.6 (0.9 to 9.4)
Rwanda	2.4 (1.6 to 3.4)	4.5 (1.3 to 8.5)	3.9 (0.6 to 8.1)
Saint Kitts & Nevis	5.2 (3.4 to 7.2)	9.3 (2.9 to 18.0)	8.3 (1.5 to 17.2)

Saint Lucia	6.3 (4.2 to 8.8)	11.3 (3.5 to 21.5)	10.0 (1.7 to 20.6)
Samoa	2.1 (1.3 to 3.1)	3.9 (1.1 to 7.5)	3.4 (0.5 to 7.1)
Sao Tome & Principe	2.6 (1.7 to 3.6)	4.7 (1.4 to 8.9)	4.2 (0.8 to 8.5)
Saudi Arabia	8.3 (5.6 to 11.1)	14.5 (4.5 to 27.2)	12.9 (2.4 to 26.5)
Senegal	3.8 (2.6 to 5.2)	6.9 (2.2 to 12.8)	6.1 (1.0 to 12.4)
Serbia	6.3 (4.2 to 8.7)	11.2 (3.5 to 21.0)	10.0 (1.6 to 20.4)
Seychelles	3.1 (2.0 to 4.4)	5.7 (1.7 to 11.0)	5.0 (0.9 to 10.4)
Sierra Leone	2.4 (1.6 to 3.3)	4.4 (1.3 to 8.3)	3.9 (0.7 to 8.0)
Singapore	5.8 (3.2 to 8.8)	10.5 (2.9 to 20.8)	9.3 (1.5 to 20.3)
Slovakia	5.6 (3.8 to 7.6)	10.1 (3.0 to 18.8)	8.9 (1.5 to 18.3)
Slovenia	5.2 (3.5 to 7.2)	9.3 (2.9 to 17.5)	8.3 (1.4 to 17.1)
Solomon Islands	3.0 (1.9 to 4.3)	5.5 (1.6 to 10.5)	4.9 (0.9 to 10.0)
South Africa	6.1 (4.1 to 8.4)	10.9 (3.2 to 20.6)	9.7 (1.7 to 19.8)
Spain	4.2 (3.0 to 6.0)	7.9 (2.4 to 14.7)	7.0 (1.2 to 14.4)
Sri Lanka	4.7 (3.0 to 6.6)	8.5 (2.5 to 16.1)	7.5 (1.2 to 15.5)
State of Palestine	6.4 (4.3 to 8.8)	11.5 (3.6 to 21.4)	10.2 (1.9 to 20.9)
Suriname	7.0 (4.8 to 9.5)	12.4 (3.7 to 23.3)	11.1 (2.0 to 22.4)
Swaziland	4.5 (3.1 to 6.1)	8.2 (2.5 to 15.2)	7.3 (1.4 to 14.7)
Sweden	3.8 (2.5 to 5.2)	6.9 (2.1 to 13.0)	6.1 (1.0 to 12.4)
Switzerland	3.9 (2.6 to 5.4)	7.1 (2.1 to 13.6)	6.2 (1.0 to 12.9)
Tajikistan	4.7 (3.1 to 6.8)	8.6 (2.7 to 16.4)	7.6 (1.3 to 15.6)
Thailand	4.0 (2.6 to 5.7)	7.3 (2.2 to 13.9)	6.4 (1.0 to 13.2)
Timor-Leste	2.9 (1.8 to 4.3)	5.4 (1.6 to 10.5)	4.8 (0.9 to 9.9)
Togo	1.6 (1.1 to 2.3)	3.0 (0.9 to 5.7)	2.7 (0.5 to 5.6)
Tokelau	1.9 (1.1 to 2.7)	3.4 (1.0 to 6.7)	3.0 (0.5 to 6.4)
Tonga	2.9 (1.8 to 4.1)	5.3 (1.6 to 10.2)	4.7 (0.9 to 9.7)
Trinidad and Tobago	6.1 (3.9 to 8.6)	10.9 (3.3 to 21.0)	9.7 (1.9 to 19.9)
Tunisia	4.9 (3.2 to 6.8)	8.9 (2.8 to 16.9)	7.8 (1.3 to 16.2)
Turkey	4.9 (3.2 to 6.9)	8.9 (2.6 to 16.8)	7.9 (1.4 to 16.4)
Tuvalu	4.4 (3.0 to 6.2)	8.0 (2.5 to 15.2)	7.1 (1.3 to 14.6)
Uganda	0.9 (0.6 to 1.3)	1.7 (0.5 to 3.3)	1.5 (0.3 to 3.2)
Ukraine	3.2 (2.1 to 4.6)	5.9 (1.8 to 11.4)	5.2 (0.9 to 10.6)
United Arab Emirates	6.6 (4.5 to 8.9)	11.7 (3.6 to 22.0)	10.4 (1.8 to 21.5)
United Kingdom	5.8 (3.9 to 7.8)	10.3 (3.1 to 19.3)	9.1 (1.7 to 18.5)
United Republic of Tanzania	1.1 (0.7 to 1.6)	2.0 (0.6 to 3.8)	1.8 (0.3 to 3.7)
United States of America	6.4 (4.3 to 8.6)	11.4 (3.5 to 21.3)	10.1 (1.6 to 20.6)
Uruguay	3.7 (2.4 to 5.2)	6.7 (2.0 to 12.7)	5.9 (1.1 to 12.2)
Uzbekistan	3.1 (1.9 to 4.6)	5.8 (1.8 to 11.3)	5.1 (0.9 to 10.7)
Vanuatu	1.3 (0.8 to 2.0)	2.5 (0.7 to 4.9)	2.2 (0.4 to 4.7)
Venezuela (Bolivarian Republic of)	5.17 (3.3 to 7.1)	9.1 (2.7 to 17.2)	8.1 (1.4 to 17.0)
Viet Nam	4.1 (2.7 to 5.8)	7.5 (2.2 to 14.3)	6.6 (1.2 to 13.6)
Zambia	3.6 (2.4 to 5.0)	6.6 (2.0 to 12.3)	5.8 (1.0 to 11.8)
Zimbabwe	4.4 (2.8 to 6.1)	7.9 (2.3 to 14.9)	7.0 (1.2 to 14.3)
